# Seasonality in land–ocean connectivity and local processes control sediment bacterial community structure and function in a High Arctic tidal flat

**DOI:** 10.1093/femsec/fiad162

**Published:** 2023-12-18

**Authors:** Eleanor R Handler, Sebastian D J Andersen, Rolf Gradinger, Maeve McGovern, Anna Vader, Amanda E Poste

**Affiliations:** Department of Arctic and Marine Biology, UiT – The Arctic University of Norway, Framstredet 39, 9019 Tromsø, Norway; Department of Arctic Biology, The University Centre in Svalbard, P.O. Box 156, 9171 Longyearbyen, Norway; Norwegian Institute for Water Research, Fram Centre for High North Research, Hjalmar Johansensgate 14, 9007 Tromsø, Norway; Department of Arctic and Marine Biology, UiT – The Arctic University of Norway, Framstredet 39, 9019 Tromsø, Norway; Department of Arctic Biology, The University Centre in Svalbard, P.O. Box 156, 9171 Longyearbyen, Norway; Norwegian Institute for Water Research, Fram Centre for High North Research, Hjalmar Johansensgate 14, 9007 Tromsø, Norway; Department of Arctic and Marine Biology, UiT – The Arctic University of Norway, Framstredet 39, 9019 Tromsø, Norway; Department of Arctic and Marine Biology, UiT – The Arctic University of Norway, Framstredet 39, 9019 Tromsø, Norway; Norwegian Institute for Water Research, Fram Centre for High North Research, Hjalmar Johansensgate 14, 9007 Tromsø, Norway; Department of Arctic Biology, The University Centre in Svalbard, P.O. Box 156, 9171 Longyearbyen, Norway; Department of Arctic and Marine Biology, UiT – The Arctic University of Norway, Framstredet 39, 9019 Tromsø, Norway; Norwegian Institute for Water Research, Fram Centre for High North Research, Hjalmar Johansensgate 14, 9007 Tromsø, Norway; Norwegian Institute for Nature Research, Fram Centre for High North Research, Hjalmar Johansensgate 14, 9007 Tromsø, Norway

**Keywords:** Arctic, bacterial community, functional diversity, land–ocean interactions, seasonal dynamics, tidal flat

## Abstract

Climate change is altering patterns of precipitation, cryosphere thaw, and land–ocean influxes, affecting understudied Arctic estuarine tidal flats. These transitional zones between terrestrial and marine systems are hotspots for biogeochemical cycling, often driven by microbial processes. We investigated surface sediment bacterial community composition and function from May to September along a river–intertidal–subtidal–fjord gradient. We paired metabarcoding of *in situ* communities with *in vitro* carbon-source utilization assays. Bacterial communities differed in space and time, alongside varying environmental conditions driven by local seasonal processes and riverine inputs, with salinity emerging as the dominant structuring factor. Terrestrial and riverine taxa were found throughout the system, likely transported with runoff. *In vitro* assays revealed sediment bacteria utilized a broader range of organic matter substrates when incubated in fresh and brackish water compared to marine water. These results highlight the importance of salinity for ecosystem processes in these dynamic tidal flats, with the highest potential for utilization of terrestrially derived organic matter likely limited to tidal flat areas (and times) where sediments are permeated by freshwater. Our results demonstrate that intertidal flats must be included in future studies on impacts of increased riverine discharge and transport of terrestrial organic matter on coastal carbon cycling in a warming Arctic.

## Teaser

The gradient from High Arctic freshwater rivers to marine fjord systems was reflected in distinct bacterial diversity and substrate utilization patterns, highlighting the significance of land–ocean interactions for microbial community structure and function in such systems.

## Introduction

Arctic rivers deliver ~4200 km^3^ of freshwater to the Arctic Ocean annually (Haine et al. 2015). These rivers act as links between terrestrial and marine systems, bridging the boundary between land and sea by delivering freshwater and terrestrial material from upstream catchment areas. Arctic rivers can carry a high volume of fine particulate organic and inorganic matter, including material originating from glacial erosion and permafrost thaw (Schreiner et al. 2014, Overeem et al. 2017, Wild et al. 2019). Much of this particulate matter is often deposited very close to the shoreline (MacDonald et al. 1998, Weslawski et al. 1999, Jong et al. 2020). Riverine dissolved organic matter can also be removed rapidly from the water column as salinity changes increase flocculation and binding with inorganic sediments (Meslard et al. 2018, Lasareva et al. 2019, Kipp et al. 2020). Where wave action is low, high rates of particle deposition can create river deltas with tidal flats (Klein 1985). In particular, mud flats are common coastal features across the High Arctic, where environmental limitations on vegetation growth often prevent tidal marshes from establishing (Church and Ryder 1972, Martini et al. 2019). These Arctic estuarine tidal flats house a range of invertebrate macrofauna and can be important feeding grounds for migratory shorebirds (Weslawski et al. 1999, Brown et al. 2012, Churchwell et al. 2018). Globally, tidal flats are among the most highly productive (Heip et al. 1995, Underwood and Kromkamp 1999) and widespread coastal ecosystems (Wang et al. 2002). Their sediments often contain a combination of terrestrially derived organic matter (Terr-OM, transported through rivers), marine detritus, and organic matter (OM) from *in situ* biological processes (Volkman et al. 2000, Wang et al. 2002, Cole et al. 2007). Yet Arctic nearshore environments, shaped by riverine inputs, remain understudied, both due to traditional divisions between terrestrial, freshwater and marine science and the logistical challenges of accessing these shallow regions (Jong et al. 2020, Klein et al. 2021).

Particulate and dissolved Terr-OM has traditionally been considered largely refractory, with limited availability for microbial degradation or food web uptake (Kattner et al. 1999, Mann et al. 2016, McGovern et al. 2020). However, recent work has identified certain portions of this pool, which may be highly bioavailable, including Terr-OM mobilized through glacial melt and permafrost degradation (Hood et al. 2009, Vonk et al. 2013, Mann et al. 2015). Increasing glacial melt, permafrost thaw, and precipitation with climate change are expected to lead to higher riverine discharge and a subsequent increase in the influx of Terr-OM to coastal systems (Christiansen et al. 2005, Haine et al. 2015, Parmentier et al. 2017, Hanssen-Bauer et al. 2019, McCrystall et al. 2021). In addition, coastal erosion is an increasingly important driver for delivery of Terr-OM and sediments to marine systems (Fritz et al. 2017). In coastal systems, riverine inputs interact with marine processes to shape nutrient dynamics, OM availability, stratification, light availability, and temperature (Mann et al. 2016, Torsvik et al. 2019, McGovern et al. 2020). Sources of freshwater shift throughout the Arctic melt season, progressing from snow melt to glacial melt and precipitation-driven run-off, with impacts on riverine biogeochemistry and receiving coastal systems (Nowak and Hodson 2015, Koziol et al. 2019, McGovern et al. 2020). At the coast, these terrestrial inputs enter a seasonally dynamic Arctic marine system, and the combination of the two processes can shape coastal Arctic microbial communities, as they respond to both physicochemical changes and shifts in OM availability (Kellogg et al. 2019, Thomas et al. 2020, Delpech et al. 2021). Furthermore, Arctic rivers can alter coastal microbial communities through delivery of allochthonous terrestrial and freshwater microbial taxa into coastal environments (Hauptmann et al. 2016, Morency et al. 2022).

With a high input of OM and strong seasonal biogeochemical gradients in tidally influenced sediments, tidal flats play a key role in global biogeochemical cycling, including cycling of carbon, nitrogen, and sulfur (Epstein 1997, Alongi 1998, Jassby et al. 2002). Microbial communities in these regions also shape the transformation and fate of terrestrial OM and nutrients, affecting the degree to which these inputs impact marine systems further offshore. For example, denitrification in estuarine tidal flats reduces nitrate loading from rivers to coastal oceans (Trimmer et al. 1998, Cabrita and Brotas 2000). Furthermore, mineralization of OM deposited in shallow sediments can release key nutrients for primary producers to the water column (Zou et al. 2016, Mougi 2020).

Bacterial communities are structured by environmental conditions, and in particular by biogeochemical gradients (Baas-Becking 1934, Fierer 2017). Differences in community composition can in turn strongly impact functional capacity (Strickland et al. 2009, Fierer et al. 2012). In tidal flat sediments, environmental gradients are both vertical, through sediment depth, and horizontal, across the surface sediments. Microbial communities and their functional capacity show strong patterns with sediment depth as oxygen concentrations decrease, interlinked with diagenesis (Köpke et al. 2005, Wilms et al. 2006, Böer et al. 2009). Estuarine tidal flats can be divided laterally into three main regions with distinct environmental conditions that shape their ecology: the supratidal, intertidal, and subtidal regions (Reineck and Singh 1980). Salinity differences along the supra tidal to subtidal gradient can be important for structuring microbial communities (Lv et al. 2016, Zhang et al. 2017, Niu et al. 2022). Community composition can also be affected by nitrogen loading, sulfate concentration, pH, and phosphorous concentration, and is often linked with seasonality (Zhang et al. 2017, Yan et al. 2018, Guo et al. 2021, Mohapatra et al. 2021, Niu et al. 2022). Sediment grain size and porosity can further shape benthic bacterial communities (Dale 1974, Probandt et al. 2017). Sea ice presence and breakup in Arctic tidal flat areas can strongly impact sediment redistribution and deposition in the intertidal zone, and decreases in ice presence and sediment freezing with climate change could lead to strong changes in the colonization patterns (McCann and Dale 1986, Węsławski et al. 2011). A better understanding of the environmental factors that drive surface sediment communities, where most degradation of organic molecules occurs (Kristensen et al. 1995, Holmer 1999), is key for improving our knowledge of processes in estuarine tidal flats.

Here, we studied the influence of riverine inputs on surface sediment microbial communities and processes in an Arctic estuarine tidal flat, in Adventfjorden, Svalbard from May to September. The main aim of this study was to identify the influence of terrestrial runoff on the structure and function of Arctic tidal flat microbial communities throughout a melt season, from May to September. To investigate *in situ* microbial community composition, we used high-throughput sequencing of the 16S rRNA gene. Functional potential was addressed through two avenues: metabolic pathway prediction from taxonomic assignment and *in vitro* carbon-source substrate utilization experiments. We hypothesized that riverine inputs would shape microbial communities and their functions either directly through delivery of terrestrial and freshwater taxa or indirectly through changes in downstream environmental conditions. To our knowledge, this study presents the highest seasonal and spatial resolution data on microbial communities in a High Arctic estuarine tidal flat to date.

## Materials and methods

Surface sediment and porewater samples were collected monthly from May to September covering the 2021 melt season in the Adventdalen and Adventfjorden system (Table S1, Supporting Information). Adventfjorden is an inner arm of the Isfjorden complex on the West coast of Spitsbergen, Svalbard. The fjord is heavily influenced by the Adventelva river and other smaller rivers throughout the melt season (McGovern et al. 2020, Nowak et al. 2021, Walch et al. 2022). The braided Adventelva river is one of the largest rivers in Spitsbergen, with ~18% of its catchment area covered by glaciers (Ziaja 2005). Typically, the Adventelva river flows from late May to early June, until between late September and early October when it freezes (Nowak et al. 2021). A large tidal flat extends from the mouth of the river to the fjord, covering ~2.5 km^2^ and characterized by braided shallow river branches. Just beyond the delta rim, the depth of the fjord rapidly increases to ~40 m. The Adventelva river carries high sediment loads to the tidal flat and fjord, resulting in estimated sedimentation rates in July ranging from 10 g m^−2^ d^−1^ near the fjord mouth to 1000 g m^−2^ d^−1^ just beyond the edge of the tidal flat (Weslawski et al. 1999, Zajaczkowski and Włodarska-Kowalczuk 2007). Previous studies have demonstrated that riverine inputs have a broad range of impacts on this estuarine ecosystem, from microbial communities in the pelagic (Delpech et al. 2021) to amphipods in the tidal flats (Skogsberg et al. 2022), though no studies have yet explored the bacterial communities directly within the tidal flats.

### Field sampling

**Figure 1.**
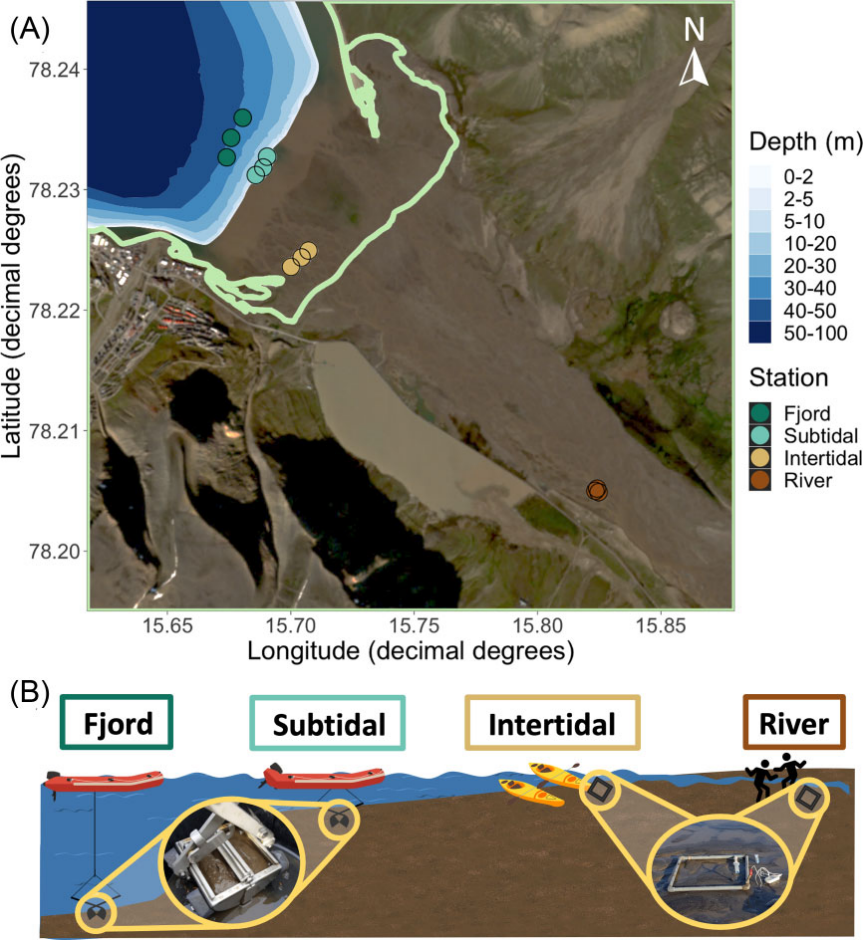
(A) Targeted sampling sites within each station throughout the season. Green line demarcates medium high tide level (Norwegian Mapping Authority). Satellite image from 14 August 2021 retrieved from Sentinel EO Browser. High frequency *in situ* NIVA operated sensors were located ~100 m upstream from the river station and 200 m toward shore from the fjord station. Exact coordinates for each month varied slightly, see Table S1 (Supporting Information). (B) Illustration of sample collection. Two distinct approaches were used to define the individual sampling areas—sediment samples were collected at the fjord and subtidal stations from a Polarcirkel boat with a 0.025 m^2^ van Veen grab with two removeable windows, while intertidal and river sampling sites were marked with a 0.25 m^2^ frame on the sediment surface.

To investigate the impact of riverine inputs on sediment microbial communities, four contrasting sampling stations were established covering the gradient from freshwater to marine conditions in Adventdalen and Adventfjorden: river, intertidal, subtidal, and inner fjord stations (Fig. 1A). Within each sampling station, surface sediment samples and porewater were collected from three sites. In the heterogeneous braided river and tidal flat, sampling sites were selected based on three main criteria: sediment ridge edges adjacent to river channels, fine grain size, and accessibility. Fine sediments were targeted to facilitate comparison with fjord and subtidal stations. In the inner fjord, sites were located in proximity to a seasonally deployed mooring with *in situ* water quality sensors operated by the Norwegian Institute for Water Research (NIVA). Intertidal and subtidal samples were collected near low tide as the tide was rising.

At river and intertidal sites, a 0.25 m^2^ frame was used to demarcate the sampling area, while for subtidal and fjord stations, sediments were sampled from the windows of a 0.025 m^2^ van Veen grab (Fig. 1B). For all campaigns aside from May, prebleached (0.5% NaClO, 20 min) and MilliQ-rinsed standard plastic spoons were used to collect ca 100 ml from the top 1 cm of sediments in a sterile Whirl-Pak plastic bag. In May, sediment samples were collected using prebleached cut-tip BD Plastipak 100 ml syringes (Becton Dickinson Norway AS, Oslo, Norway).

Porewater was extracted in the field from the top two centimeters of sediments with a Rhizon CSS (Rhizosphere Research, Wageningen, The Netherlands). Approximately, 30–100 ml of porewater was collected from each station in a BD Plastipak 60 ml acid washed (15% HCl by volume, 24 h, well-rinsed with deionized water) plastic syringe (Becton Dickinson Norway AS) and stored in acid washed and precombusted (4.5 h, 450ºC) brown glass bottles.

River water was collected each month for use with Biolog EcoPlates^TM^, using a clean plastic bucket. Water temperature was measured *in situ* with a handheld thermometer. Salinity, conductivity, and pH were measured either in the field or immediately upon return to the University Centre in Svalbard (UNIS) using a multiparameter meter (HI 9829, Hanna Instruments, USA). The pH sensor was calibrated prior to every sampling campaign.

All sediment and water samples were kept cool and dark until further processing at UNIS, within 8 h of collection.

### Sample processing

Utensils for splitting sediment samples were prebleached (0.5% NaOCl for 20 min) between sampling days. The sediments in each Whirl-Pak were homogenized with a metal spoon, split with the spoon and a spatula into subsamples, and preserved for analyses. Both utensils were sprayed with ethanol and burned between each sample. For DNA extraction, three subsamples were frozen at −80ºC in sterile 2 ml cryo vials. For photopigment concentrations, one subsample was frozen at −80ºC. Preweighed bottles of known volume were filled with sediment and frozen at −20ºC for bulk density, porosity, grain size, and loss on ignition (LOI) measurements. For one site from each station, subsamples were stored for a maximum of 2 h in the dark at 4ºC for Biolog EcoPlates^TM^.

As the nominal pore size of the Rhizon CSS is reported by the manufacturer to be 0.18 µm, porewater was not additionally filtered upon return from the field. Subsamples, ~5–20 ml, for characterization of chromophoric dissolved organic matter (cDOM) in porewater were stored in acid-washed, precombusted glass bottles. Porewater samples for analysis of dissolved inorganic nutrient concentrations were preserved with 4 N H_2_SO_4_ (1%–2% final concentration by volume) and stored in acid-washed, precombusted glass bottles. Silicate concentrations were expected to be sufficiently high that potential contamination from storage in glass bottles would be negligible. All porewater samples were stored at 4ºC in the dark until analysis.

### Laboratory analyses

#### Physico-chemical characteristics of sediment and porewater

Porosity was calculated using a wet–dry method (Zaborska et al. 2008). Organic content of sediments was determined through LOI for 5 h at 450ºC (Sutherland 1998, Wang et al. 2011, Morata et al. 2020). Grain size distributions were determined by wet sieving through mesh sizes of 2 mm, 1 mm, 500 µm, 250 µm, 125 µm, and 63 µm (Bale and Kenny 2005). Following extraction with acetone for 24 h, sediment chlorophyll-*a* (chl-*a*) was measured fluorometrically with a Turner 10-AU fluorometer (Turner Designs, USA) calibrated with pure algal chl-*a* (Sigma-Aldrich, Oslo, Norway). Samples were then acidified with 2–3 drops of 10% HCl, and fluorescence was measured again to determine phaeopigment and acid-corrected chl-*a* concentrations (as in Parsons et al. 1984).

To characterize cDOM in porewater, absorbance was measured at 1 nm intervals from 200 to 900 nm using a Shimadzu UV-1900 UV–Vis spectrophotometer (Shimadzu Corporation, Tokyo, Japan) within 4–7 days following sample collection. Raw absorbance values were processed according to McGovern et al. (2020) to calculate the absorption coefficient at 254 nm, spectral slopes from 275 to 295 nm and 350 to 400 nm and the ratio between them (slope ratio). The ratio between absorption coefficients at 250 and 365 nm (E2/E3) was also calculated according to De Haan and De Boer (1987). Absorption at 254 nm is generally positive related to DOC concentrations, while spectral slopes from 275 to 295 nm and 350 to 400 nm, slope ratio, and E2/E3 are typically negatively related to molecular weight of DOM (Hansen et al. 2016).

Due to low sample volumes, porewater salinity was measured using a salinity refractometer (Magnum Media Salinity 10ATC), after storage in acid-washed and burned sealed glass bottles for up to 6 months. Concentrations of inorganic nutrients in porewater were measured at the Norwegian Institute for Water Research (NIVA, Oslo, Norway) using inductively coupled plasma mass spectrometry (as in Kaste et al. 2022).

#### Microbial community structure and function

Microbial DNA was extracted from 0.4 to 1.6 g wet weight of sediment using the PowerSoil® DNA Isolation Kit (MO BIO Laboratories Inc., Carlsbad, CA, USA) following kit instructions. Extraction blanks (MilliQ water) were included with each batch and sequenced. PCR tests using bacterial primers 515F (Parada et al. 2016) and 806R (Apprill et al. 2015) were performed following each extraction for quality control—amplification was never observed in extraction blanks. Library preparation, including amplification, and sequencing (Illumina MiSeq 2 × 300 bp paired-end V3 chemistry) were performed by the Integrated Microbiome Resource (IMR, Dalhousie University in Halifax, Nova Scotia, Canada) using standard protocols (Comeau et al. 2017). Sequences in the V3–V4 region of the 16S rRNA gene were amplified using primers 341F (CCTACGGGNGGCWGCAG) and 805R (GACTACHVGGGTATCTAATCC) (Illumina/Klindworth et al. 2013). The resulting sequences are available in NCBI's Sequence Read Archive under project accession number PRJNA1054200.

Biolog EcoPlates^TM^ (Biolog Inc., Hayward, CA) were used to assess potential microbial community function under different salinity treatments related to utilization of a range of carbon substrates (Garland and Mills 1991, Insam 1997). EcoPlates are 96-well plates that contain three replicates of 31 different carbon sources, chosen to differentiate between community-level physiological profiles (Insam 1997), and three blank wells with no substrate. All nonblank wells contain a tetrazolium salt that turns purple with bacterial respiration in the well, i.e. when bacteria metabolize the provided carbon substrate (Garland and Mills 1991).

Following each sampling event from June through September, one sample from each station was used to inoculate plates within 8 h of sample collection (Table S2, Supporting Information). Water for sediment suspensions was first filtered through 0.2 µm polycarbonate syringe filters to remove all organisms. Suspensions were made with 1.8 ml of sediment diluted to 1:272 with treatment water using two steps of sonication and dilution to ensure visibility in the final wells. To investigate microbial activity throughout tidal cycle conditions, sediment samples from the inter- and subtidal flat were suspended in three different types of water: river water from Adventelva river (collected during the main sampling campaign), filtered seawater from Adventfjorden (taken from the UNIS sea water supply, with an intake pipe at a depth of ~30 m), and a 1:3 mix of seawater to river water to simulate brackish water. River sediments were only suspended in river water and fjord sediments were only suspended in filtered seawater. Both river water and filtered seawater were analyzed for dissolved inorganic nutrients in the same manner as porewater samples and had fairly similar nutrient concentrations, with higher nitrate/nitrite and phosphate in the marine water and higher silicate in the river water (Figure S1, Supporting Information).

Each well of an EcoPlate was inoculated with 140 µl of sediment suspension, and the absorbance of each well at 590 nm was recorded immediately with a Multiskan GO spectrophotometer (Thermo Fisher Scientific, USA). Inoculated plates were incubated in the dark at 10ºC in a Termaks cooling incubator (Nino Labinteriör AB, Kungsbacka, Sweden). The absorbance at 590 nm was then recorded for each well daily for 14 days to assess community substrate utilization. In May, only fjord sediments suspended in seawater were used to inoculate an EcoPlate which was incubated at 4ºC. Results from May were used for comparison, but not included in formal analysis. EcoPlate data and environmental data are available at the Northeastern University Digital Repository Service (Handler 2023b).

### Data processing and analysis

All processing of DNA sequences and EcoPlate absorbances and statistical analyses were performed within the R framework (v4.1.0; R Core Team 2021), using RStudio (RStudio Team 2021). The *tidyverse* ecosystem was used throughout data processing and analyses (Wickham et al. 2019). R scripts are available online (Handler 2023a).

#### DNA sequence processing

We received demultiplexed sequences from IMR. Primers were first clipped using cutadapt (v3.7, Martin 2011). Sequences were then processed with DADA2 (Callahan et al. 2016), using a pipeline modified after Pearman et al. (2021). Taxonomy of the resulting amplicon sequence variants (ASV) was assigned using the RDP Naive Bayesian Classifier algorithm (Wang et al. 2007) against the SILVA SSU nonredundant (v 138.1) reference database (Quast et al. 2012), with a minimum bootstrap of 70. ASVs classified as chloroplasts or mitochondria were removed, and the results were combined to form a *phyloseq* object (McMurdie and Holmes 2013). Contaminants identified from sequencing of extraction blanks were removed from the dataset using *decontam* (Davis et al. 2018). Finally, only ASVs with more than one sequence in more than two samples were kept, removing 3.7% of reads retained up to that step (Table S3, Supporting Information). Samples with fewer than 3000 reads were not used for downstream analyses, removing one May intertidal sample and one July subtidal sample.

#### Biolog EcoPlate data processing

Absorbance values at 590 nm were adjusted for blanks and initial readings of each well according to Sofo and Ricciuti (2019). The area under the curve, which condenses several kinetic measures into one metric, was calculated for each substrate on each plate using OD_i_ values (Hackett and Griffiths 1997). For each plate, four metrics were used to estimate diversity of substrate use: substrate richness, average well color development (AWCD), Shannon's diversity index, and Simpson’s diversity index. With an OD_i_ greater than 0.250, wells were considered purple, i.e. the bacterial community was able to use that substrate (Garland 1996, Sofo and Ricciuti 2019). Substrates were only considered to have been utilized by the community if at least two of the three replicate wells on a plate turned purple. Substrate richness was measured as the number of substrates utilized on a plate at the final time point (day 14). AWCD provides a metric of the development of the plate and was calculated by taking the mean OD_i_ of all wells across each plate (excluding blanks) at the final time point. Shannon’s and Simpson’s diversity indices were calculated according to Zak et al. (1994).

#### Statistical analysis

To investigate spatial and seasonal trends in environmental conditions across samples, principal component analysis (PCA) was performed on scaled environmental variables. Highly skewed variables were natural-log transformed prior to z-scaling. All ordinations were made using the *vegan* package (Oksanen et al. 2022).

To evaluate alpha diversity, all samples were rarefied with random subsampling to 4130 reads, the lowest number of reads in any sample, using the packages *phyloseq* (McMurdie and Holmes 2013) and *microbiome* (Lahti and Shetty 2012–2019). Alpha diversity estimators, the number of ASVs, Chao1 (Chao 1984), Abundance-based Coverage Estimator (ACE; Chao and Lee 1992), Shannon’s and Inverse Simpson’s diversity indices, and Pielou’s evenness index, were calculated for each sample with rarefied, non-normalized, and standardized (proportions by sample multiplied by median read count) datasets using *phyloseq*.

Shared ASVs between stations were evaluated on the rarefied dataset, using the package *MicrobiotaProcess* (Xu and Yu 2021) and plotted with the package *VennDiagram* (Chen and Boutros 2011). All ASVs that had been found at relative abundance greater than 0.05% in any river sample were identified as riverine taxa, and their relative abundances were calculated for all other samples to determine the contribution of riverine taxa.

For investigations of beta diversity, the community dataset was transformed to proportions and treated as compositional (Gloor et al. 2017). The community matrix was Chi-square transformed and Euclidean distances were calculated using the *vegan* package. Hierarchical cluster analysis was performed on the distance matrix, using Ward’s clustering criterion (1963), and plotted with the *dendextend* package (Galili 2015). Other ordinations and hierarchical clustering based on non-normalized, standardized, rarefied, Hellinger transformed, and clr-transformed data using Bray–Curtis dissimilarity or Euclidean distances showed similar patterns.

Abundant genera in each cluster were identified by grouping ASVs by genus and calculating means of proportional abundances within each cluster. Indicator taxa for each cluster were determined with Dufrêne–Legendre Indicator Values, using the multipatt function of the *indicspecies* package (De Cáceres and Legendre 2009) with 999 permutations. Only ASVs with an indicator value > 0.7 and a *P*-value ≤ 0.001 were considered significant indicators, and indicator ASVs were considered highly abundant if they had a relative abundance of at least 0.5% within their cluster (Delpech et al. 2021). The taxonomic composition of highly abundant indicators was examined.

To identify potential community functions from the taxonomic assignments, we used Tax4Fun (Aßhauer et al. 2015) to predict KEGG metabolic pathway reference profiles. The KEGG pathway matrix was curated to remove functions irrelevant to bacterial communities and targeted functions were investigated with the metabolic pathways.

We used variance partitioning (Borcard et al. 1992) with testing by permutation (999) to quantify the contributions of station and month to variation in community composition. Canonical correspondence analysis (CCA) was used to examine the relationships between environmental variables and microbial community structure. Environmental variables were treated in the same manner as for PCA above and were then grouped by sediment characteristics, porewater chemistry, and indicators of OM quality. Constraining variables within each group were selected using supervised forward and reverse model selection with the ordistep function in *vegan*. To avoid collinearity, we chose to focus on large and small fractions for grain size, rather than the intermediates, and chl-*a* rather than phaeopigment concentrations. Selected variables from each group were then combined to a single model, which was similarly evaluated. Significance of each variable was subsequently tested with a permutation test (*n* = 999). Multicollinearity of variables, tested using vif.cca in the *vegan* package after ordination, showed low rates of collinearity in the final model. Spearman correlations of indicator taxa abundance with the same environmental variables were calculated with the function rcorr in the *Hmisc* package (Harrel 2019).

Plots were created with the *ggplot2* package (Wickham 2016). Maps were made with the *PlotSvalbard* package (Vihtakari 2020). Due to low sample size, to test for differences between groups, for diversity metrics, proportions of riverine taxa in other samples, environmental variables, and EcoPlate results, we used the Kruskal–Wallis rank sum test (Kruskal and Wallis 1952) with Dunn’s *post hoc* test (Dunn 1964) using the *dunn.test* package (Dinno 2017), with *P*-values corrected as described in Benjamini and Hochberg (1995).

## Results

### Environmental context

In general, biogeochemical characteristics of sediments and porewater showed similarities between the river and the intertidal stations, as well as between the fjord and subtidal stations (Figure S2, Supporting Information). All variables measured varied between stations and across months, though they exhibited different trends. Measured sediment temperatures ranged from 0.5 to 8.6ºC, with fjord sediments consistently coldest and July and August as the warmest months. While sediment oxygen concentrations were not explicitly measured, we did not observe evidence of anoxic conditions in any of the sampled surface sediments, including in the deeper sediments collected with the van Veen grab.

Sediments were generally finer in the fjord and subtidal and coarser in the river and intertidal (Figure S3, Supporting Information). Sediment silt and clay content generally increased throughout the melt season. Organic content and porosity also generally increased seasonally (Figure S4, Supporting Information), and the two were highly correlated (Spearman’s rho = 0.73, *P* < 0.001). Porosity ranged from 41% (intertidal in May) to 77% (fjord in August), while organic content ranged from 0.7% (river in July) to 6.8% (fjord in August).

Porewater chemistry differed between stations and months (Figure S5, Supporting Information). Porewater was consistently fresh in the river and the intertidal from June and July (median 1.5 PSU). In May, intertidal porewater salinity was hypersaline (44.5 PSU), and it was brackish in August and September (6.5 PSU). Subtidal and fjord porewater salinity was “marine” (37.5 PSU), except in July when the median subtidal porewater salinity was 19.5 PSU. We did not observe a strong influence of tidal cycle on salinity, likely because the intertidal and subtidal were primarily sampled at low to rising tide. It should be noted that porewater samples were stored before analysis, so these values are likely overestimates of *in situ* salinity due to evaporation. Concentrations of most inorganic nutrients in porewater did not show pronounced seasonal variation. Phosphate and ammonium concentrations followed similar patterns to porewater salinity, respectively ranging from medians of 0.1 µmol l^−1^ and 1 µmol l^−1^ in the river to 0.4 µmol l^−1^ and 71 µmol l^−1^ in the fjord. Porewater nitrate and nitrite concentrations ranged widely (0.07–38.9 µmol l^−1^) and did not show clear seasonal or spatial patterns. Across all samples, silicate concentrations in porewater were generally high (mean ± sd: 62 ± 35 µmol l^−1^).

Indicators of OM quality showed strong seasonal and spatial patterns (Figure S6, Supporting Information). Chl-*a* concentrations ranged widely (0.035–15 µg ml^−1^), with generally higher proportions of phaeopigments in sediments with lower chl-*a* concentrations. Chl-*a* concentrations were consistently lowest in the river (median 1.3 µg ml^−1^). The highest chl-*a* concentrations were found in fjord sediments in June and July (median 5.6 µg ml^−1^), with fairly low proportions of phaeopigments (28%). cDOM absorption coefficients at 254 nm were high in the fjord (median 11) and low in the river and intertidal (3 and 5), with more seasonal variation in the subtidal (5 in June and July to 13 in August and September). The subtidal shifted from low values in June and July (5) to higher values in August and September (13). E2/E3 ratios of cDOM absorption were high in May (9.2), both in the fjord and intertidal. Throughout the melt-season, E2/E3 ratios were generally highest in the subtidal (9.4), with lower values from the river (7.2), intertidal (5.3), to fjord (3.6). cDOM spectral slopes from 275 to 295 nm and 350 to 400 nm followed the same pattern as E2/E3 ratios.

### Community composition

#### Alpha diversity

Following processing of sequences and reads, including removal of singletons, a total of 7131 ASVs were identified across all samples. Bacterial diversity varied somewhat between stations. Estimated richness (Chao1) was higher in fjord and subtidal sediments than in river and intertidal sediments (Figure S7, Supporting Information; Kruskal–Wallis test (KW): *P* = 0.047). However, observed richness (number of ASVs), evenness, and Shannon’s diversity index were not significantly different between stations, (Figure S7, Supporting Information; KW: *P* = 0.48, 0.84, and 0.84). Similar patterns for richness (observed, Chao1, and ACE), diversity (Shannon’s and Inverse Simpson’s), and evenness (Pielou’s) metrics were found when they were calculated with the rarefied, non-normalized, and standardized datasets (Figure S7, Supporting Information).

#### Taxonomic composition

**Figure 2.**
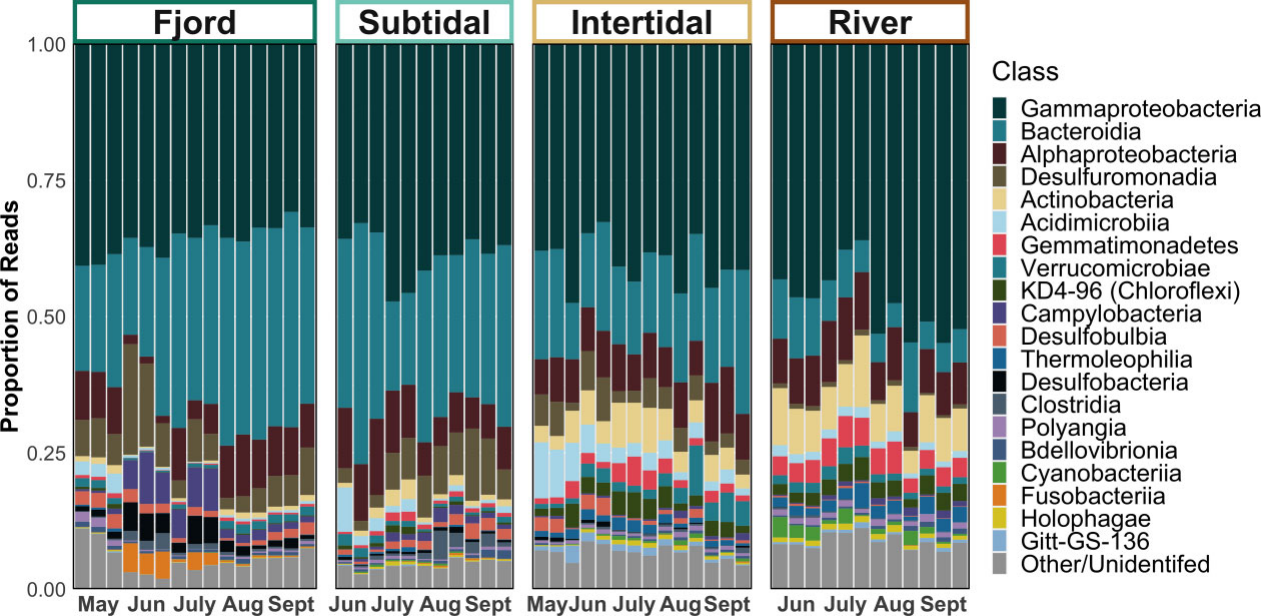
Proportions of reads within the most represented bacterial classes. The top 20 most represented classes are displayed here, ordered by their overall proportional abundance across all samples. Samples are separated by sampling station and ordered by month within each station.

Gammaproteobacteria was the most represented class in all stations with mean relative read abundance across all samples of 40 ± 6% (Fig. 2). Fjord, subtidal, and intertidal communities were also dominated by Bacteroidia (mean read abundances of 30%, 29%, and 17% respectively), though these were much less prominent in riverine sediments (8%). Desulfuromonadia displayed a similar pattern (7%, 7%, 4%, and 0.7%), while Alphaproteobacteria had a more stable read abundance of 8 ± 3% across all samples. Both Actinobacteria and KD4-96 (a clade within Chloroflexi) had higher read abundances in the river and intertidal than the subtidal and fjord (6.5% vs. 1.1% and 3% vs. 0.4%, respectively). In general, we found strong similarities between communities within the three replicates collected at each station in each month.

Across the whole season, the fjord had the highest number of unique ASVs (1312) (Figure S8, Supporting Information). The subtidal had the least with 249, and the intertidal and river had 432 and 468 unique ASVs, respectively. A total of 2473 ASVs were found in at least three of the stations, and 83% of the 2733 riverine ASVs were also found in other stations, especially in the intertidal (71%) as compared with the subtidal (58%) and fjord (43%).

#### Seasonal and spatial variation in bacterial community structure

Microbial community composition in sediments was significantly correlated with both station and sampling month, based on permutation tests (*P* = 0.001). Results of variance partitioning on correspondence analysis showed that station accounted for 26% of community variation and month accounted for 12%, with no variation explained by both factors (all proportions significant at *P* = 0.001).

**Figure 3.**
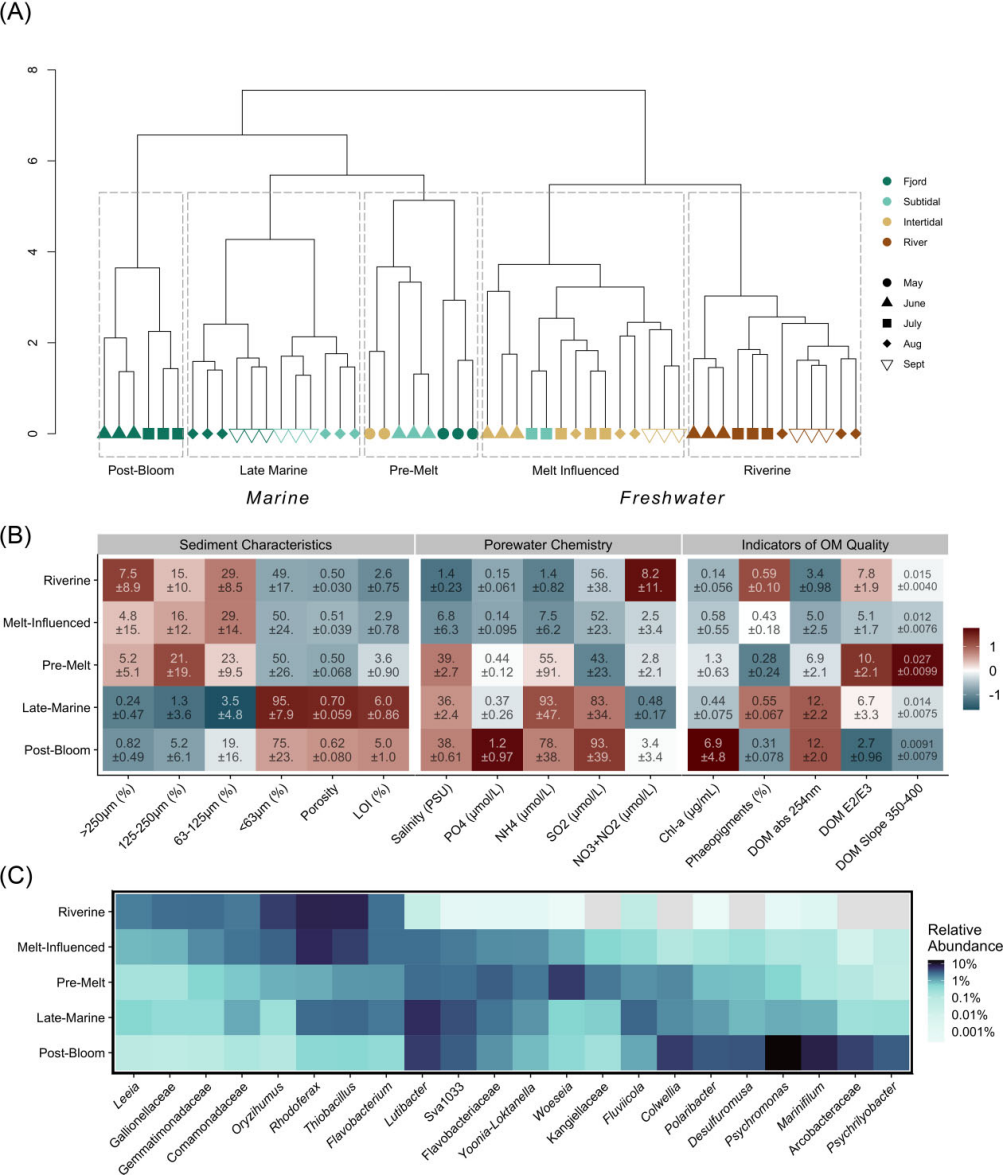
(A) Hierarchical clustering of microbial communities, using Ward’s clustering criterion on chi-squared distances between samples. (B) A heatmap displaying medians of z-scaled environmental variables for each cluster. Text within fields show unscaled means with standard deviations. Red indicates relatively high values while blue indicates relatively low values. Kruskal–Wallis tests showed significant differences between clusters for all environmental variables (*P* < 0.01) except for porewater nitrate and nitrite and porewater silicate. See Appendix for results of Dunn’s *post hoc* tests for pairwise comparisons (Tables S4–S6, Supporting Information). (C) A heatmap showing mean relative abundance of abundant genera (defined as contributing > 2% of the total reads in any cluster) from each cluster, colored on a log-scale for increased resolution. Light blue indicates low relative abundance while dark purple indicates high relative abundance. Light gray tiles indicate no reads.

Results of hierarchical clustering on community composition showed two distinct groups, separating marine and freshwater communities (Fig. 3A). Low porewater salinity and cDOM absorption at 254 nm distinguished freshwater from marine conditions (Fig. 3B; Dunn’s *post hoc* test (D): *P* < 0.01). These groups were further divided into five significantly different clusters (Anosim *post hoc* all *P* = 0.001), which were named based on physical and temporal characteristics of their environments: *Riverine, Melt-Influenced, Pre-Melt, Late-Marine, and Post-Bloom*. The two clusters within the freshwater group, *Riverine* (all river samples) and *Melt-Influenced* (all intertidal samples excluding May, as well as July subtidal samples), were further characterized by coarse, compacted sediments with relatively low values for porosity and sediment organic content, and low concentrations of ammonium and phosphate. The only significant differences between the two freshwater clusters were higher cDOM E2/E3 ratios in the *Riverine* cluster and higher salinity in the *Melt-Influenced* cluster (D: *P* = 0.03, 0.02). Though not statistically significant, chl-*a* concentrations were also higher and phaeopigments lower in the *Melt-Influenced* than the *Riverine* cluster.

Within the marine group, we identified three clusters: *Pre-Melt, Late-Marine*, and *Post-Bloom*. The *Pre-Melt* cluster, with all May samples and June subtidal samples, was similar in sediment characteristics to the freshwater clusters, but had higher cDOM E2/3 ratios than the *Melt-Influenced* cluster (D: *P* = 0.001), and higher cDOM slope 350–400 nm and ammonium and phosphate concentrations than both freshwater clusters (D: *P* < 0.02). The *Late-Marine* and *Post-Bloom* clusters exhibited different sediment characteristics from the others, with finer grains and higher LOI and porosity (D: Tables S4–S6, Supporting Information). They also had high concentrations of ammonium and silicate and high cDOM absorption at 254 nm. The *Late-Marine* cluster included fjord and subtidal communities from August and September. The *Post-Bloom* cluster, with fjord communities from June and July, was distinct from all others but the *Pre-Melt* in its high chl-*a* concentrations (D: *P* < 0.01).

The most abundant genera varied between the clusters, though some were widely abundant (Fig. 3C and Table 1). *Rhodoferax, Oryzihumus*, and *Thiobacillus* were abundant in both *Riverine* and *Melt-Influenced* communities, with the latter two abundant in *Late-Marine* as well. *Lutibacter* was abundant in all clusters other than *Riverine*. The *Pre-Melt* and *Post-Bloom* clusters had the least similar abundant genera to the other clusters. In addition to *Lutibacter*, the *Post-Bloom* communities were dominated by *Psychromonas, Marinifilum, Colwellia*, and an unidentified genus of Arcobacteraceae, while the most abundant genera in the *Pre-Melt* communities were *Woeseia*, an unidentified genus of Flavobacteriaceae, an unidentified genus of Sva1033, and *Yoonia-Loktanella*. Sva1033 was also abundant in *Late-Marine* communities. Indicator taxa analysis identified further distinctions between the clusters, with indicator taxa from both the abundant taxonomic groups and those less well represented overall (Table 1).

**Table 1.**
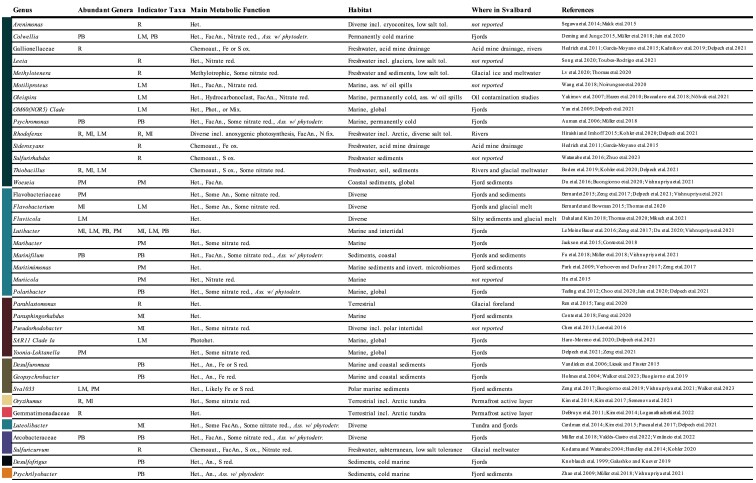
Metabolic, habitat, and location information for genera identified as top five most abundant in any cluster or abundant indicator taxa for any cluster. Taxa are grouped by class, colored bar on the left corresponds to colors in Fig. 2. All taxa are aerobic unless otherwise listed. Abbreviations: R = Riverine, MI = Melt-Influenced, PM = Pre-Melt, LM = Late-Marine, PB = Post-Bloom; Het. = heterotrophic, Chemoaut. = chemoautotroph, Phot. = phototroph, Mix. = mixotroph, Photohet. = photoheterotroph, FacAn. = facultative anaerobe, and An. = anaerobe.

**Figure 4.**
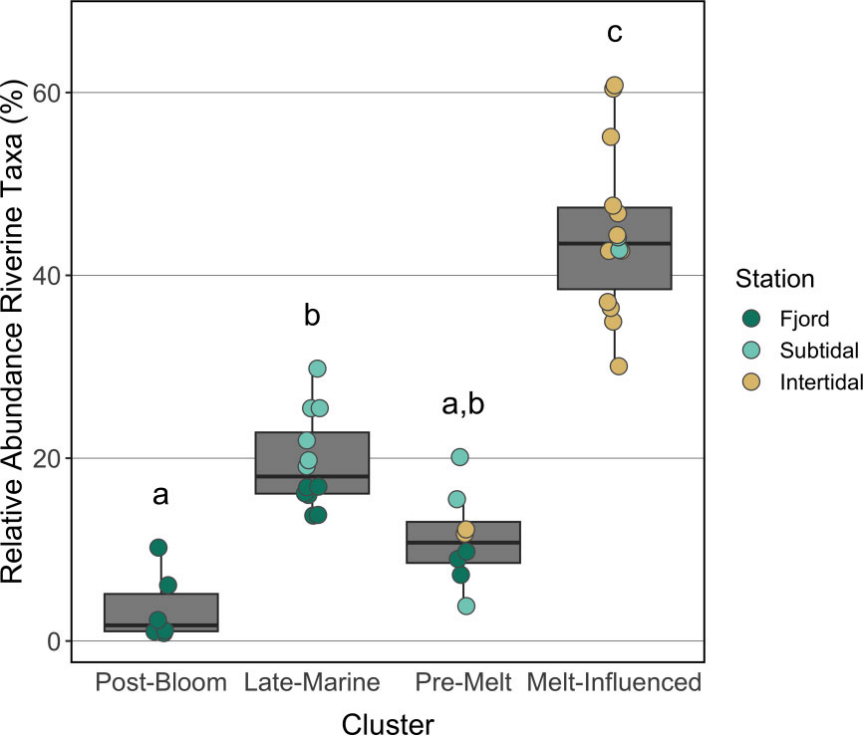
Proportional abundance of riverine taxa in other community clusters. Riverine taxa were identified as ASVs with at least 0.05% relative abundance in any river sample. Points are individual samples, colored by sampling location. Kruskal–Wallis test showed significant differences between clusters (*P* < 0.001), lowercase letters along the *x*-axis indicate significant differences between clusters (D: *P* < 0.01).

The relative abundance of riverine taxa present in communities from other clusters (Fig. 4) was highest in the *Melt-Influenced* communities (median proportion of riverine taxa of 44%), followed by *Late-Marine* communities (18%), and *Pre-Melt* communities (11%). The lowest relative abundance of riverine taxa was found in *Post-Bloom* communities (2%).

#### Potential function inferred from community composition

**Figure 5.**
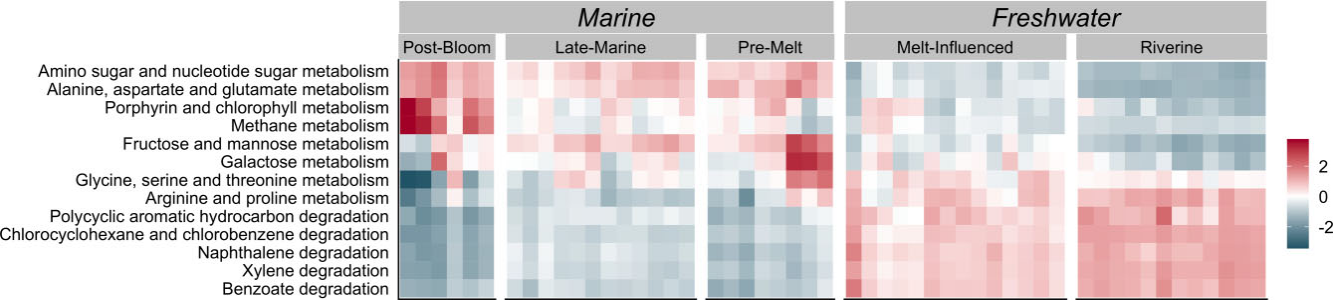
Differential abundances of selected potential metabolic and OM degradation functional capacities predicted with Tax4Fun based on taxonomic assignment. Potential functions were z-scaled for comparison across samples, and samples are grouped by cluster. Red indicates a relatively high abundance while blue indicates a relatively low abundance. See Supplementary for full results of metabolic and degradation pathways (Figure S9, Supporting Information).

Potential community functions inferred from taxonomic assignments showed clear distinctions between the freshwater and marine communities (Fig. 5, see also Figure S9, Supporting Information). Marine communities generally had higher potential capabilities for metabolism of more bioavailable molecules, including metabolisms of fructose and galactose, and the functional potential of *Pre-Melt* and *Late-Marine* communities were very similar. Freshwater communities had higher potential capacity for degradation of more recalcitrant organic compounds including aromatic molecules such as naphthalene, xylene, and polycyclic aromatic hydrocarbons.

#### Environmental drivers of community composition

**Figure 6.**
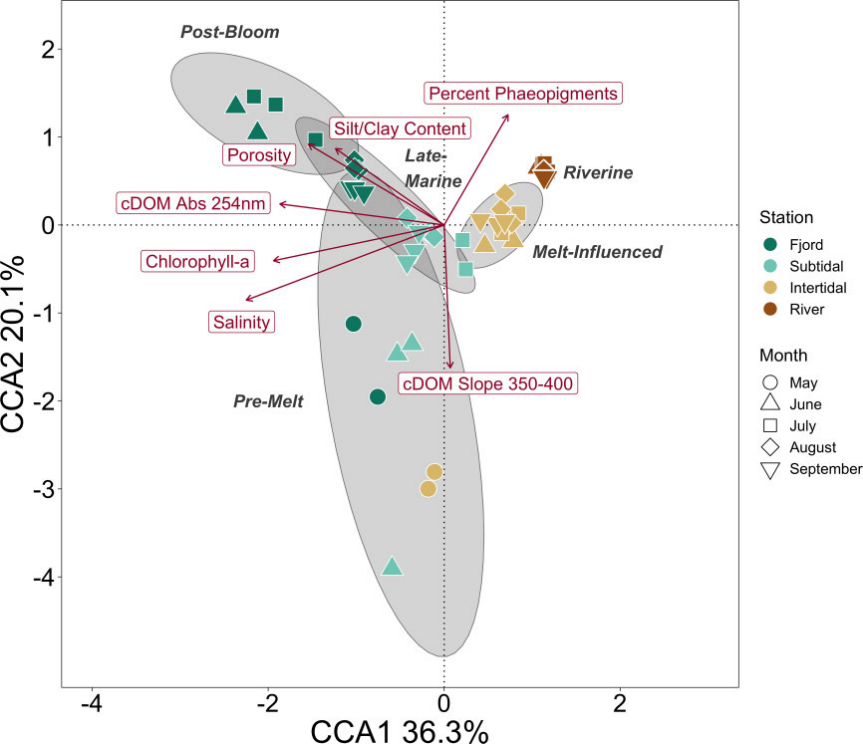
CCA with community composition and environmental variables. Points are samples, colored by station and shaped by month. Community composition was transformed to proportions and environmental variables were z-scaled before ordination. Gray ellipses represent 95% confidence intervals for clusters from hierarchical clustering analysis, labeled in gray text. Significant constraining variables are represented in red. Three samples (“5-Fjord-y”, “6-Fjord-y”, and “6-Intertidal-y”) were not included due to missing environmental data from low porewater volumes. Constrained inertia represented 45.7% of the total. Variance inflation factors for all terms were less than 6.5.

Permutation tests identified significant variables shaping community composition in the CCA model (Fig. 6). Porewater salinity, sediment chl-*a* concentrations, porosity, phaeopigments (% of total pigments), sediment silt/clay content, and cDOM absorption at 254 nm were all significant contributors to explaining the variability in community composition (*P* < 0.05). The high *F*-value of the porewater salinity model suggests that porewater salinity accounts for a large degree of variation among microbial communities, and communities accordingly separated along a salinity gradient from river to fjord on the first axis of the CCA (Fig. 6). The gradient along the first axis was also correlated with sediment porosity, the silt/clay content in sediments, the proportion of coarse material, and chl-*a* concentration in sediments. The arrangement of communities along this gradient suggests that these environmental variables are important for shaping communities’ differences from river to fjord. The *Pre-Melt* cluster separates from other communities along the second axis, suggesting these communities are associated with lower proportions of phaeopigments in the sediment and higher E2/E3 ratios. Similar results were found with RDA on Hellinger-transformed and clr-transformed community data (Figure S10, Supporting Information). Correlations of relative abundance of indicator taxa with environmental variables showed similar patterns, mirroring seasonal and spatial patterns in environmental characteristics (Figure S11, Supporting Information).

### Carbon substrate utilization depends on suspension water

**Figure 7.**
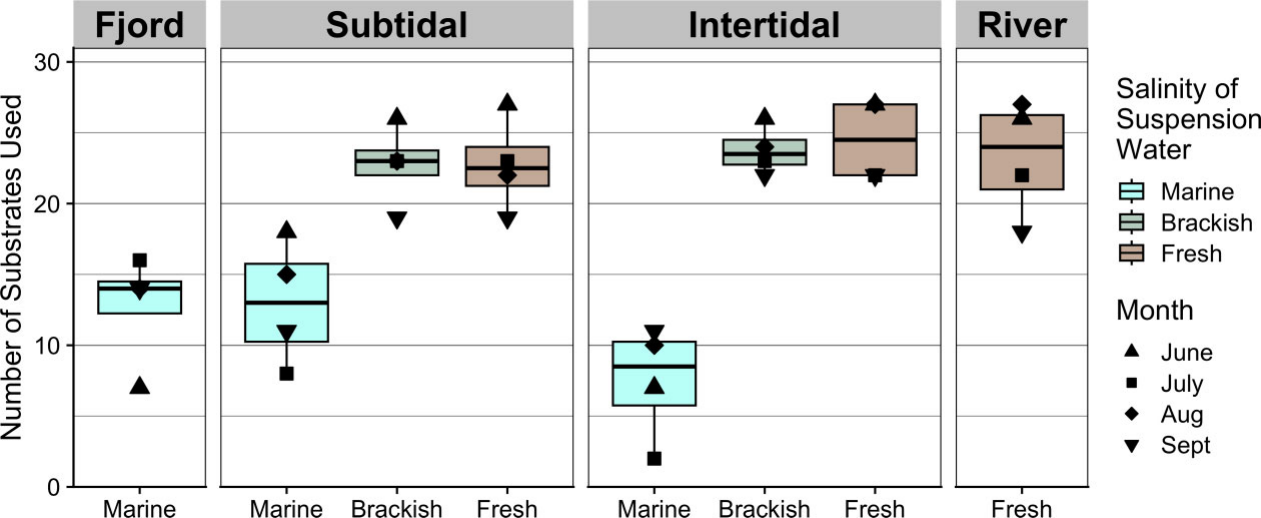
Number of substrates used in Biolog EcoPlates. Plates are grouped by sediment sampling location and type of water used for sediment suspensions. Black points represent single plates, with shape depicting the sampling month. Kruskal–Wallis test showed significant differences between salinity treatments (*P* < 0.0001), with Dunn’s *post hoc* test showing no difference between fresh and brackish suspensions while both were different from marine (*P* < 0.001).

Microbial communities used between 2 and 27 substrates on each plate, with the lowest number of substrates used by July intertidal communities suspended in marine water and the highest number of substrates utilized in June subtidal/intertidal and August river/intertidal communities suspended in river water (Fig. 7). Most communities, regardless of suspension water, used all available polymers and certain carbohydrates. There was little change in carbon substrate utilization throughout the melt season. Furthermore, in May prior to river melt, fjord sediments suspended in marine water (incubated at 4ºC rather than 10ºC) showed similar patterns in substrate use to the marine suspensions throughout the melt-season (Figure S12, Supporting Information).

Across all stations and months, microbial communities were capable of utilizing a larger number of substrates when suspended in fresh or brackish water (medians 22.5 and 23 substrates used) than when suspended in marine water (median 11 substrates used, Fig. 7, D: *P* < 0.001). Similar patterns were observed using AWCD, Shannon’s diversity index, and Inverse Simpson’s diversity index (Figure S13, Supporting Information).

Some substrates were used consistently by communities suspended in fresh or brackish water but not by communities suspended in marine water, regardless of sediment origin (Figure S14, Supporting Information). These included three of the five available amino acids four of eight carboxylic acids, one of two amines, one of two phenolic compounds, and two of 10 carbohydrates. Only threonine was used more often in marine compared to brackish and freshwater suspensions, and then only by fjord communities.

Microbial communities suspended in fresh or brackish water used similar substrates, regardless of station (Figure S14, Supporting Information). However, communities suspended in marine water showed a difference in substrates used based on station, with more substrates used by fjord and subtidal communities (medians 14 and 13) than by intertidal communities (8.5).

## Discussion

### Melt water transports allochthonous taxa downstream

Taxa known to occur in the permafrost active layer, glacial systems, or acid mine drainage were identified in all riverine communities, suggesting a high degree of connectivity between the catchment and riverine sediments. While many of the abundant genera in the *Riverine* communities have previously been found in the Adventelva river and other Arctic freshwater systems, Gemmatimonadaceae and *Oryzihumus* are more frequently associated with terrestrial soil systems including Arctic tundra and the active layer of permafrost on Svalbard (Table 1). As the nonglaciated area of Adventdalen is covered by 90% permafrost (Humlum et al. 2003), these taxa may have been transported to the river from catchment soils. Acidobacteria are well-documented members of Arctic tundra soils, including in the active layer of Svalbard permafrost (Männistö et al. 2013, Schostag et al. 2015, Xue et al. 2020). It was, therefore, unexpected to find such low abundances in this study, although low Acidobacteria abundances have also been reported in other Svalbard freshwater systems (Wang et al. 2016, Kosek et al. 2019). Many indicator taxa for the *Riverine* communities have previously been identified in connection with Svalbard glaciers (Table 1). Glacier-associated organisms are likely carried downstream with meltwater, settling in sediments along the way. While often considered neutrophilic, iron oxidizing *Riverine* indicator taxa from Gallionellaceae and *Sideroxyans* (Hedrich et al. 2011) have been identified in high abundances in acid mine drainage near Longyearbyen (García-Moyano et al. 2015) and in eastern Siberia (Kadnikov et al. 2019).

As melt sources and riverine biogeochemistry shift throughout the season (Nowak and Hodson 2015, Koziol et al. 2019, McGovern et al. 2020), we expected to find changes in riverine taxa as the melt season progressed. While clustering analysis showed distinctions between June and July–September riverine communities, overall, riverine communities were seasonally stable. One main exception was *Aquaspirillum* (*arcticum* group), which was only found in relatively high abundances in June. This group can be very abundant in snow in the High Arctic (Harding et al. 2011), suggesting early snow melt in the catchment transported these cells to riverine communities.

Riverine taxa were found in all other communities, from the intertidal zone to the fjord system, suggesting downstream transport. They were most abundant in the *Melt-Influenced* cluster, indicating a high degree of connectivity between the river and the *Melt-Influenced* communities in the tidal flats. Similarly, allochthonous riverine taxa have previously been found in Isfjorden surface waters and sediments (Delpech et al. 2021) and in estuaries elsewhere in the Arctic (Hauptmann et al. 2016, Kellogg et al. 2019).

Transport of riverine taxa might confer new functional capacities in downstream communities. Riverine taxa observed in the current study possessed capabilities for a diverse range of biogeochemical processes, including degradation or incorporation of organic compounds, carbon fixation through the oxidation of sulfur or iron, anoxygenic photosynthesis, and nitrate reduction. While some cultured members of these groups tolerate wide ranges of salinity, others are known to have low salt tolerances (Table 1). It is likely that some riverine taxa were unable to grow when deposited in the marine environments of the fjord and subtidal. However, in the *Melt-Influenced* communities, low salinities suggest that transported bacteria were likely more readily able to survive and grow, with potential implications for the fate of terrestrially derived carbon and nutrients at the river–fjord interface.

### Combination of riverine inputs and local processes shape bacterial communities

Environmental variables, linked to both seasonality and spatial variability, were strongly associated with differences in community composition, as shown in other systems (Baas-Becking 1934, Fierer 2017). Porewater salinity was the most important factor for shaping community composition, as previously observed in temperate tidal flats (Lv et al. 2016, Zhang et al. 2017, Niu et al. 2022). However, while previous studies suggest nutrient concentrations can be important for shaping microbial communities in tidal flats (Yan et al. 2018, Mohapatra et al. 2021, Niu et al. 2022), none of the measured inorganic nutrients in porewater were identified as significant predictors of community composition in this study. This was likely due to fairly high concentrations across samples, suggesting all nutrients were available in sufficient quantities to not be limiting. In addition to salinity, microbial communities were largely shaped by physical properties of the sediments (grain size and porosity) and by the quantity and quality of OM (chl-*a* concentration, cDOM absorption at 254 nm, and cDOM spectral slope, correlated with molecular weight with lower weight considered higher quality), as found in pelagic systems (Sipler et al. 2017, Kellogg et al. 2019). Some of the variation in these significant variables is directly linked to riverine influx (e.g. salinity), while others can be attributed to seasonal marine processes, like high concentration of chl-*a* following a phytoplankton bloom.

#### *Melt-Influenced* environment and communities

Flushing of tidal flat sediments, porewater, and microbial communities with high riverine discharge during snowmelt likely caused the dramatic shift in environmental conditions and bacterial communities as the melt season began. May intertidal and June subtidal bacterial communities grouped together in the *Pre-Melt* cluster, dominated by characteristic marine taxa previously found in Svalbard fjords (Table 1). With the onset of the melt season, freshwater discharge from the river flushed out the intertidal (May–June), and later subtidal (June–July), sediments, decreasing porewater salinity, cDOM absorption at 254 nm, and cDOM spectral slope, indicating lower molecular weight. The *Melt-Influenced* bacterial communities responded to these seasonal environmental changes, becoming dominated by the river-associated genera *Rhodoferax, Thiobacillus*, and *Oryzihumus*, with the addition of marine and generalist taxa that are found in a range of habitats. The combination of freshwater and marine organisms in Arctic tidal flats has also been observed in macrofaunal communities (Churchwell et al. 2016). Two strains of *Rhodoferax* were identified as indicator taxa for the *Melt-Influenced* cluster, suggesting that some freshwater taxa can adapt to the more dynamic tidal flat environment.

Recent work has suggested that microbial community functional capacity can be correlated with the chemodiversity of available OM (Rivers et al. 2013, Berggren and Giorgio 2015, Ruiz-González et al. 2015). Arctic riverine systems often have a high proportion of complex Terr-OM (Behnke et al. 2021), and Svalbard glacial-fed rivers are no different (McGovern et al. 2020, Kellerman et al. 2021). The high potential capacity for degradation of more complex organic molecules found in *Riverine* and *Melt-Influenced* communities likely reflects the diversity of available OM (also in upstream terrestrial and freshwater source areas). This suggests that complex Terr-OM deposited in their environments might be degraded by the bacterial communities.

Differences in environmental conditions between the *Melt-Influenced* and *Riverine* clusters were likely driven by local processes and, in turn, shaped community composition and potential function. For example, higher chl-*a* concentrations in the *Melt-Influenced* cluster might have been driven by microphytobenthos, found locally in intertidal regions of the Adventfjorden tidal flat (Wiktor et al. 2016), or by deposited algae from riverine inputs, with either process increasing the available OM in the sediments. Correspondingly, we found higher relative abundances of heterotrophic bacteria in these communities than in the river, which was more dominated by autotrophic taxa (Table 1). Nearly all abundant indicator taxa were members of heterotrophic genera, largely from groups known to tolerate a wide range of environmental conditions (Table 1). For example, *Luteolibacter*, an indicator taxon, has been associated with degradation of complex carbohydrates and aging phytoplankton in Svalbard fjords (Cardman et al. 2014, Wietz et al. 2021). The higher relative abundance and importance of heterotrophic taxa for distinguishing the *Melt-Influenced* communities could suggest an increased importance of degradation of OM in these communities as compared to riverine communities. With low rates of sedimentation in the intertidal flats (Weslawski et al. 1999) limiting OM burial, the intertidal and occasionally subtidal flats could be important areas for heterotrophic bacteria during the melt season, driving remineralization of OM, and release of carbon dioxide and inorganic nutrients to the water column (and atmosphere).

#### *Post-Bloom* environment and communities

While the subtidal and intertidal reflected the influence of freshwater inflow transitioning into June and July, the fjord communities followed a very different trajectory, likely shaped by the deposition of phytodetritus following phytoplankton blooms. Elevated chl-*a* concentrations were detected in inner Adventfjord in May (Andersen 2022), and the high chl-*a* concentrations in the *Post-Bloom* cluster (fjord sediments in June and July) was likely a result of phytoplankton settling. All abundant indicator taxa for these communities were strongly positively correlated with chl-*a* concentrations in the sediments, and many of the abundant prokaryotic genera and indicator taxa have been found to increase with additions of phytodetritus in experimental studies (Table 1). This was further reflected in functional predictions, showing distinctly high potential for degradation of porphyrin and chlorophyll. Community shifts with addition of phytodetritus have been found across a range of benthic ecosystems, from the deep sea to coastal sediments (Franco et al. 2007, Tait et al. 2015, Hoffmann et al. 2017), and the distinct *Post-Bloom* communities are likely a result of this type of fertilization from fjord processes, rather than a downstream response to riverine influence. Many of the abundant genera and indicator taxa in *Post-Bloom* communities were from groups known to be either obligately or facultatively anaerobic (Table 1) with anaerobic conditions likely forming within OM aggregates in the surface sediments (Reise 1985). These taxa and conditions contribute seasonally distinct biogeochemical functional potential, including sulfur and iron reduction and OM fermentation (Table 1). Overall, this finding is in contrast with recent work in Isfjorden which found little phytodetritus driven seasonal variation in sandy coastal sediments (Miksch et al. 2021).

#### *Late-Marine* environment and communities

Riverine inputs altered fjord and subtidal environmental conditions through the deposition of sediments, although the subsequent impact this has on microbial communities remains uncertain. Previous studies have found high rates of sedimentation from riverine discharge in these regions (Weslawski et al. 1999, Zajączkowski and Włodarska-Kowalczuk 2007), and the seasonal decrease in sediment grain size, coupled with increasing porosity and organic content found in this study suggests that these samples were also impacted by sediment deposition from riverine inputs. Sediments deposited by the Adventelva river tend to be very fine (< 63 µm; Rodenburg 2019). The high correlation between fine grain size, porosity, and organic content follows similar patterns to those found in temperate tidal flats (Dale 1974, Watling 1991, Vigano et al. 2003). The abundant indicator taxa for the *Late-Marine* cluster were highly correlated with these sediment characteristics, suggesting the riverine deposits might play a role in shaping the community, as has also been found in a temperate river delta (Alvisi et al. 2019, Fazi et al. 2020). One abundant genus from this cluster, *Fluviicola*, has been found in higher abundances in silty rather than sandy environments in coastal Svalbard (Miksch et al. 2021), though the seasonality of other environmental variables likely affects its presence as well.

Overall, the bacterial communities in the *Late-Marine* cluster did not show a clear response to riverine inputs and seemed to be more influenced by local marine conditions. The *Late-Marine* communities were similar to the *Pre-Melt* communities (both dominated by *Lutibacter* and Sva1033), though certain transported riverine taxa, as discussed in more detail above, were also abundant in the *Late-Marine* communities. Two of the abundant indicator taxa for the *Late-Marine* cluster, the globally distributed marine groups SAR11 Clade Ia and the OM60 clade, have previously been identified as indicator taxa for August pelagic communities in Isfjorden (Delpech et al. 2021), suggesting a high degree of connectivity between the water column and the sediments in the late summer. Interestingly, two abundant indicator taxa from *Late-Marine* communities are associated with oil spills (Table 1). While *Motiliproteus* members can utilize a wide range of organic compounds, *Oleispira* are hydrocarbonoclasts with a highly limited range of substrates they use for growth (Table 1). High volumes of boat traffic in Adventfjorden (over 1800 port calls in 2019, Port of Longyearbyen 2023) might contribute to accumulation of hydrocarbons in the sediments—with previous studies reporting concentrations of polycyclic aromatic hydrocarbons in sediments (collected in August) from Adventfjorden that were up to 16 times higher than expected background levels (Holte et al. 1996). Functional predictions for the *Late-Marine* communities showed a high capacity for degradation of more simple or bioavailable molecules, such as amino sugars, glucose, and fructose, which are often available in marine systems (Benner and Kaiser 2003, Davis and Benner 2005). The dominance of heterotrophic taxa in the *Late-Marine* cluster indicates high potential for utilization of deposited OM (Table 1). However, with elevated functional potential for degradation of highly bioavailable sugars and amino sugars over more complex compounds, these communities may be more capable of utilizing fresh marine detritus rather than Terr-OM.

### Potential functional capacity may not be realized under local environmental conditions

Potential community functions, predicted from taxonomic assignments, may not be realized in *in situ* conditions. While Tax4Fun showed distinctly different functional potential between clusters, these are based on DNA relative abundances, which may not reflect active communities, especially in these dynamic regions with high potential for passive transport of bacterial cells (First and Hollibaugh 2010, Sun et al. 2020, Breitkruez et al. 2021).

Experimental incubations using Biolog EcoPlates^TM^ (Insam 1997) provided insight into realized community functioning in different salinity environments. Despite strong differences in community composition, little variation in functional capacity was observed seasonally or spatially. However, across all microbial communities, a higher number of substrates were consistently used in fresh and brackish suspensions than in marine suspensions, even when community composition remained the same. This was unexpected, as previous work has found tradeoffs with salinity increases—with utilization of some substrates decreasing while utilization of others increase (Chen et al. 2017)—and lower functional diversity in Arctic freshwater than marine pelagic communities (Tam et al. 2003). The consistency of the 11 additional substrates utilized in fresh and brackish suspensions was also unexpected given the high degree of variability in community composition between samples. The degree to which microbial community functional capacity is realized is known to be shaped by environmental conditions (Strickland et al. 2009, Fierer et al. 2012), and this study confirms that this paradigm also applies to Arctic tidal flat communities, where salinity is likely a dominant factor. Given that many cultured members of riverine-associated genera exhibit low salt tolerances (Table 1), we suspect that transported riverine bacteria were active in the fresh and brackish intertidal and subtidal incubations, while they were inactive in the marine salinity incubations. To our knowledge, this is the first study of tidal flat sediment whole community functional profiles to use a range of salinities for suspension water. Our findings suggest that future work in tidal flats cannot ignore the high amount of variation in microbial functional capacity related to changes in salinity.

### Implications and perspectives in a warming Arctic

Our findings suggest that bacterial communities in Arctic riverine sediments are capable of degrading a wide range of complex organic molecules, like Terr-OM. However, with fairly low rates of OM deposition in riverine sediments, autotrophs more likely dominate these systems (Weslawski et al. 1999). In fjord sediments where large amounts of Terr-OM are deposited, transported riverine taxa may be inhibited by marine conditions, leaving the sediments as a site of burial for Terr-OM (Koziorowska et al. 2016, Bianchi et al. 2020, McGovern unpublished work). However, upstream in the estuarine tidal flat, communities seem to have a high potential for degradation of complex organic molecules. As salinity shifts tidally and seasonally in Arctic river deltas, they likely oscillate between acting as hotspots for processing of Terr-OM and as sites of high Terr-OM burial.

The Arctic is warming at nearly four times the rate of the global average, causing widespread impacts on Arctic landscapes and ecosystems (Rantanen et al. 2022). Precipitation, riverine discharge, and additions of Terr-OM are expected to increase across the Arctic with climate change (Haine et al. 2015, Parmentier et al. 2017, Hanssen-Bauer et al. 2019, Meredith et al. 2019). The microbial communities in estuarine tidal flats will likely be affected by these changes, as salinity regimes, sources of allochthonous taxa, and sources of Terr-OM entering the system contribute to shaping microbial communities and their functioning. Given the high potential for degradation of Terr-OM in the intertidal flat region, these nearshore areas must be considered in future studies of Arctic coastal carbon cycling.

## Conclusion

This study aimed to identify the ways in which terrestrial runoff influences the structure and function of Arctic tidal flat microbial communities throughout the melt season. We studied a single system for the duration of one melt season, but the processes identified are likely relevant for other Arctic coastal systems, especially those impacted by glacier-fed rivers. We found clear evidence that freshwater discharge shapes environmental conditions, which in turn structure microbial community composition. Porewater salinity was identified as a key factor for shaping bacterial communities, alongside physical sediment properties and OM availability and quality. Microbial communities and their biogeochemical functions were divided between the freshwater dominated intertidal and the marine dominated subtidal. Freshwater inflow is also a direct source of riverine and terrestrial taxa to downstream communities, and these taxa exhibit unique functional capacities not widely found in the Arctic marine system. However, carbon substrate utilization experiments demonstrated that environmental conditions strongly impact realized functions, with communities exhibiting higher diversity of substrate use in fresh and brackish water than in marine water regardless of community composition. Thus, future work in tidal flats must consider the impact of dynamic salinity variations on microbial community functions in space and time. In a changing Arctic, the fate of increased fluxes of Terr-OM to coastal areas remains a key knowledge gap. Positioned at the land–ocean interface, tidal flats, and the functioning of the diverse microbial communities that inhabit them, need to be considered to fully understand the impacts of a changing climate on Arctic coastal ecosystems.

## Supplementary Material

fiad162_Supplemental_File
