# Supplementary material for: Seasonality in land–ocean connectivity and local processes control sediment bacterial community structure and function in a High Arctic tidal flat
Source: FEMS Microbiol Ecol. 2023 Dec 18;100(1):fiad162. doi: 10.1093/femsec/fiad162 (PMC10799726; doi:10.1093/femsec/fiad162)
Supplement: fiad162_Supplemental_File [file fiad162_supplemental_file.pdf]

Supplementary Information for:

Seasonality in land-ocean connectivity and local processes control sediment bacterial community structure and function in a high Arctic tidal flat

Eleanor Ruth Handler[1,2,3], Sebastian David Junker Andersen[1,2,3], Rolf Gradinger[1], Maeve McGovern[1,3], Anna Vader[2], Amanda Poste[1,3]

[1] Department of Arctic Marine Biology, UiT – The Arctic University of Norway, Tromsø, Norway
[2] Department of Arctic Biology, The University Centre in Svalbard (UNIS), Longyearbyen, Norway
[3] Norwegian Institute for Water Research (NIVA)

Contents:

1. Supplemental Methods
    i. Processing of DNA sequences
2. Supplemental Figures
    i. Figure S1. Water chemistry for suspension water used for Biolog EcoPlates.
    ii. Figure S2. PCA with environmental variables
    iii. Figure S3. Sediment grain size distribution
    iv. Figure S4. Sediment environmental variables by station.
    v. Figure S5. Porewater chemistry by station.
    vi. Figure S6. Indicators of organic matter quality and quantity by station.
    vii. Figure S7. Alpha diversity metrics of community dataset.
    viii. Figure S8. Venn diagram with shared ASVs by station.
    ix. Figure S9. Heatmap of Tax4Fun results with all metabolism and degradation pathways.
    x. Figure S10. RDAs with Hellinger and clr transformed community data.
    xi. Figure S11. Spearman rank correlation of indicator taxa abundance with environmental variables.
    xii. Figure S12. Heatmap displaying median area under the curve (AUTC) for each substrate on Biolog EcoPlates.
    xiii. Figure S13. Alpha diversity metrics of Biolog EcoPlates.
    xiv. Figure S14. Heatmap displaying number of times used for each substrate on Biolog EcoPlates.
3. Supplemental Tables
    i. Table S1. Sediment sampling dates, precise locations, times when sampling began, and tidal information.
    ii. Table S2. Sediment samples used for Biolog EcoPlates.
    iii. Table S3. Read counts for each sample following DNA processing steps.
    iv. Table S4. Results of Dunn's post-hoc test comparing sediment characteristics between clusters.
    v. Table S5. Results of Dunn's post-hoc test comparing porewater chemistry between clusters.
    vi. Table S6. Results of Dunn's post-hoc test comparing indicators of organic matter between clusters.

Supplemental Methods

DNA processing details:

We received demultiplexed sequences from IMR. Primers were first clipped using cutadapt (v3.7, Martin 2011), with a maximum error rate of 0.1. Sequences lacking primer sequences were discarded. Sequences were then processed with DADA2 (Callahan et al. 2016), using a pipeline modified after Pearman et al. (2021).

Forward reads were truncated to 253 bp while reverse reads were truncated to 189 bp, based on visual inspection of the error profiles. The maximum numbers of "expected errors" allowed were two and five, accounting for differences in quality between the forward and reverse reads, respectively. Sequences with a quality score of 2 or less were discarded. Sequences were dereplicated for each sample and error rates for forward and reverse reads were learned with the first 10^8 bp. This parametric error matrix was used to infer amplicon sequence variants (ASVs) from the dereplicated reads, using the "pseudo-pool" option within DADA2 to reduce impact of different read numbers on diversity (Kleine Bardenhorst et al. 2022). Forward and reverse reads were merged with a minimum overlap of 12 bp and no mismatches allowed. Chimeric sequences were removed with the *removeBimeraDenovo* DADA2 function using the "consensus" method.

Taxonomy of the resulting ASVs was assigned using the RDP Naive Bayesian Classifier algorithm (Wang et al. 2007) against the SILVA SSU nonredundant (v 138.1) reference database (Quast et al. 2013), with a minimum bootstrap of 70. ASVs classified as eukaryotes, chloroplasts, or mitochondria were removed, and the results were combined to form a *phyloseq* object (McMurdie and Holmes 2013), which was used for further processing. Contaminants were identified from sequencing of extraction blanks and removed from the dataset using the prevalence method in the *decontam* package (Davis et al. 2018). Only one contaminant was identified, and it was only found in three samples at very low abundances: June-Subtidal-x (0.01%), June-River-y (0.02%), and July-Fjord-z (0.02%). Finally, only ASVs with more than one sequence in more than two samples were kept, removing 3.7% of reads retained up to that step (Table A4). Samples with fewer than 3000 reads were not used for downstream analyses, removing one May intertidal sample and one July subtidal sample.

Supplemental Figures

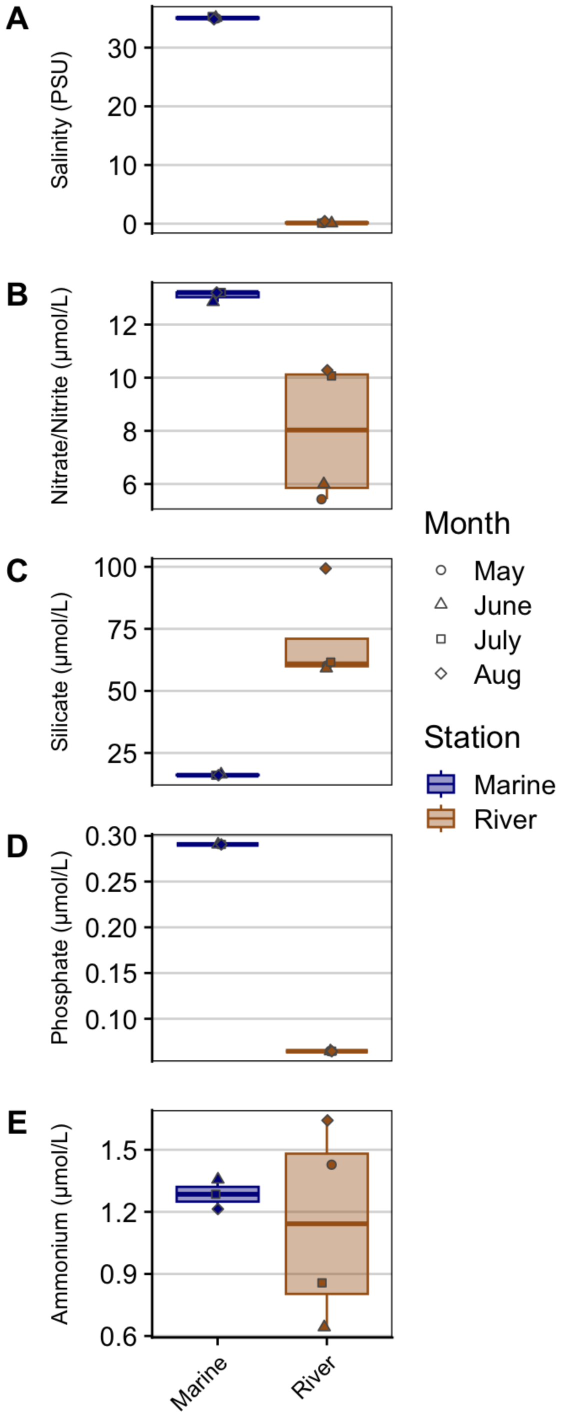

Figure S1. Boxplots displaying water chemistry for suspension water used for Biolog EcoPlates each month. Points represent individual samples with shapes denoting month. **(A)** Salinity. **(B)** Concentration of nitrate and nitrite. **(C)** Concentration of silicate. **(D)** Concentration of phosphate. **(E)** Concentration of ammonium.

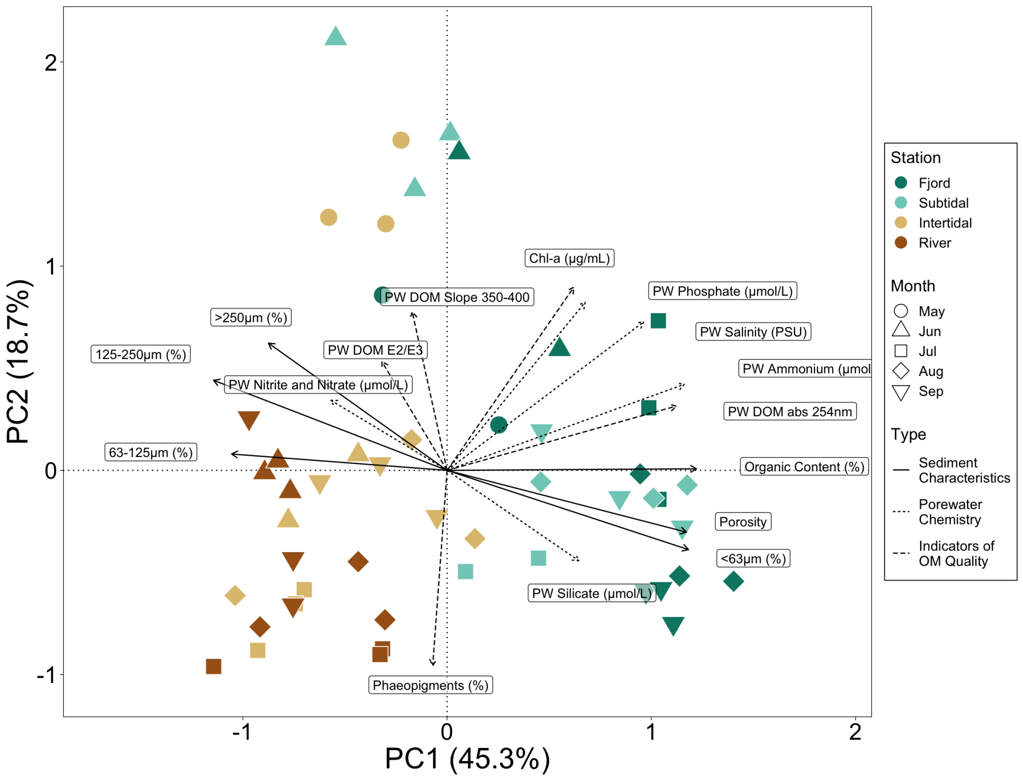

Figure S2. Principal component analysis on z-scaled environmental data. Highly skewed variables (chl-*a* concentration, inorganic nutrient concentrations, and sediment grain size fractions 125-250µm and >250µm) were natural log-transformed prior to z-scaling to increase normality. Each point is one sample. Colors represent station and shape shows month. Different line types on loading arrows display groupings of environmental variables.

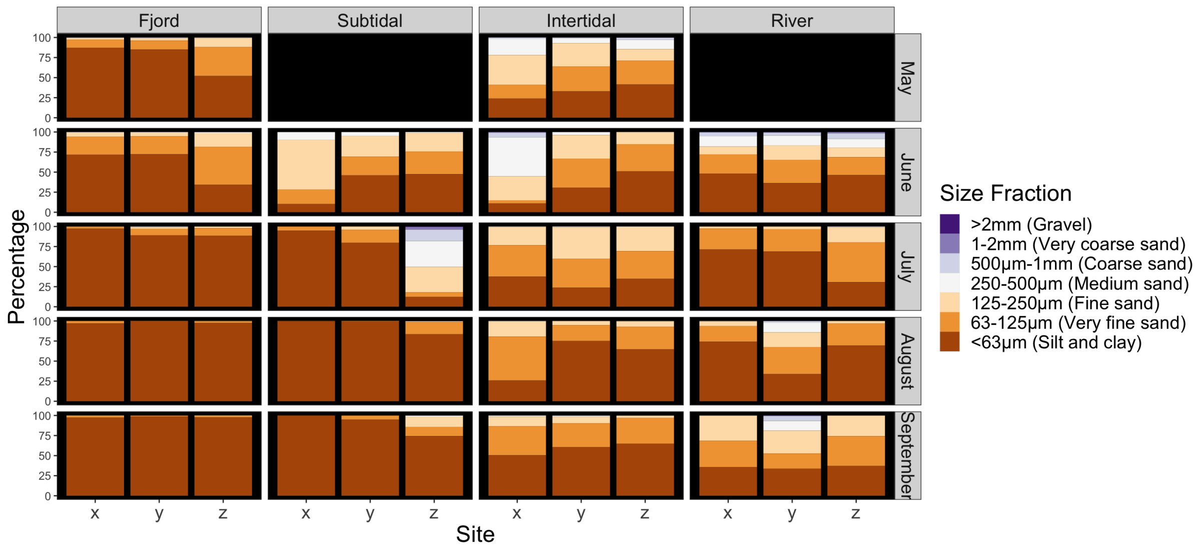

Figure S3. Distribution of grain size fractions in each sample, as determined through wet sieving. Each bar shows the distribution of a single sediment sample.

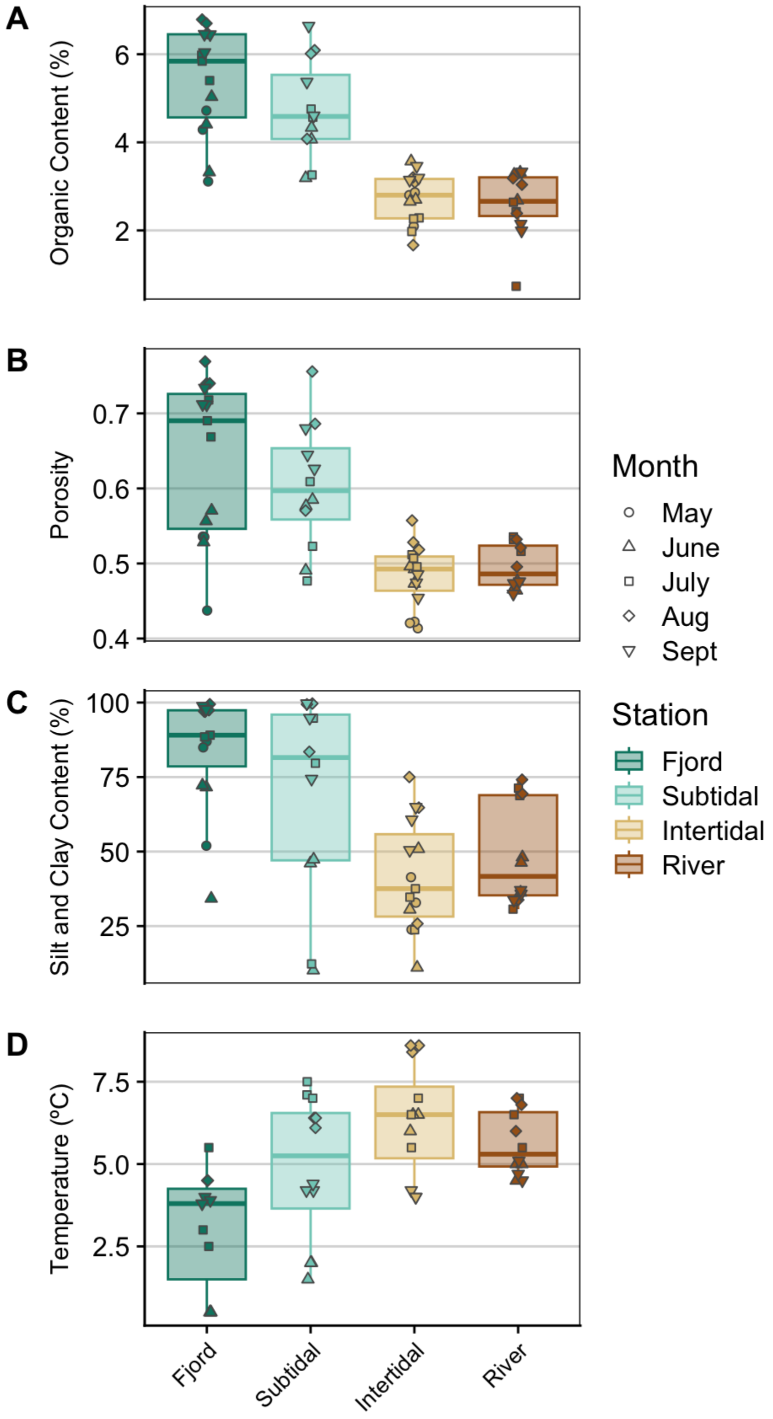

Figure S4. Boxplots displaying environmental variables for each sediment sampling site, grouped by station. Points represent individual samples with shapes denoting sampling month. **(A)** Organic matter content of sediments as determine by LOI. **(B)** Porosity of the sediment (water content by volume). **(C)** Percent by mass of the sediment less than 63µm. **(D)** Sediment temperature, not recorded in May.

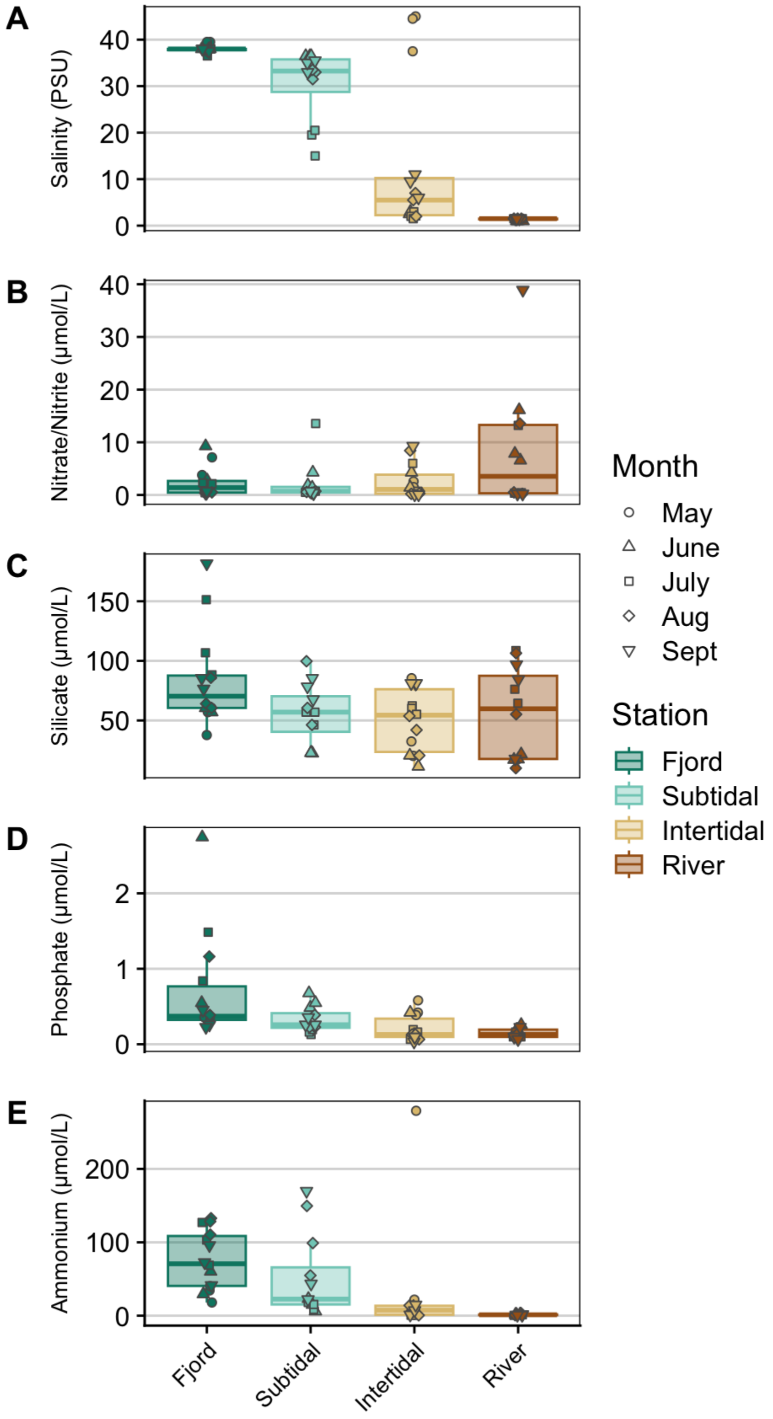

Figure S5. Boxplots displaying porewater chemistry for each sediment sampling site, grouped by station. Points represent individual samples with shapes denoting sampling month. **(A)** Salinity. **(B)** Concentration of nitrate and nitrite. **(C)** Concentration of silicate. **(D)** Concentration of phosphate. **(E)** Concentration of ammonium.

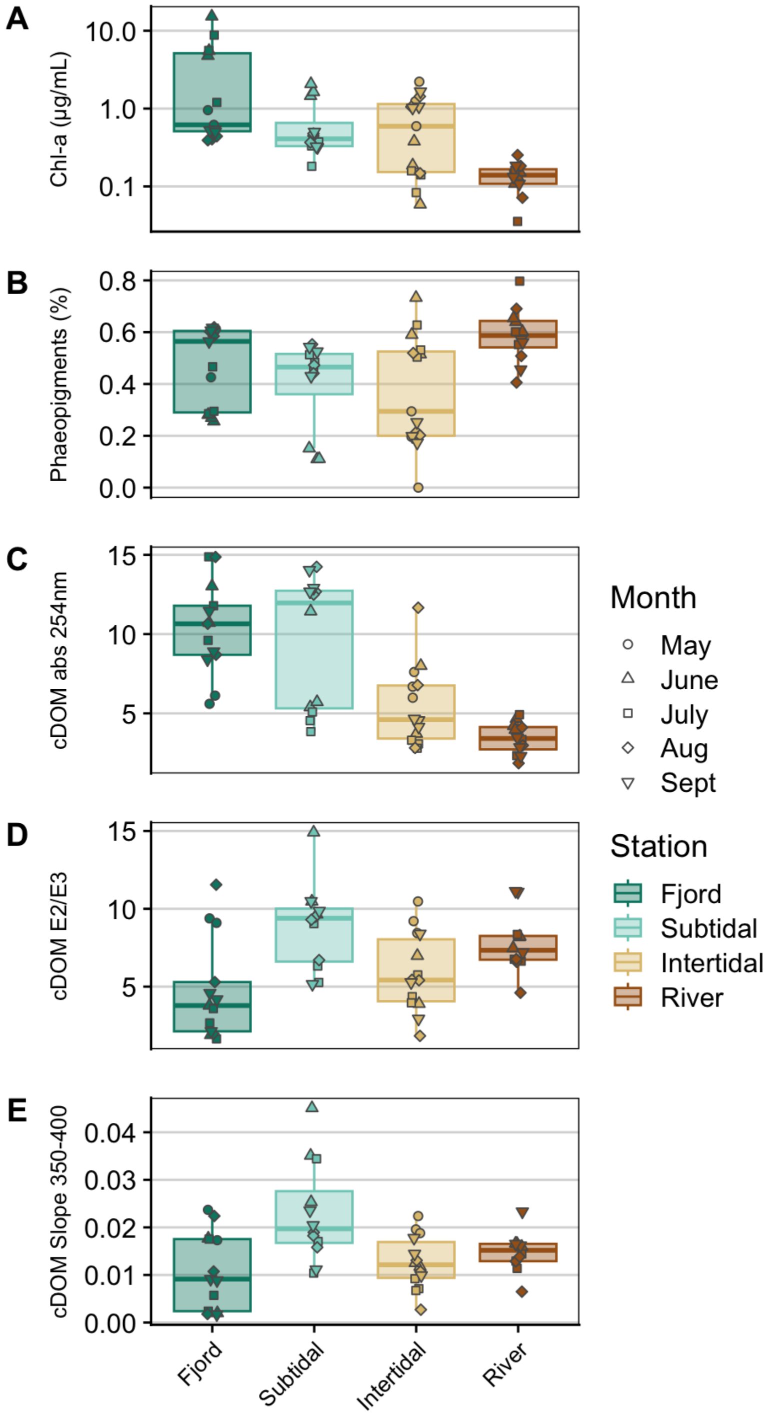

Figure S6. Boxplots displaying indicators of organic matter quality and quantity for each sediment sampling site, grouped by station. Points represent individual samples with shapes denoting sampling month. **(A)** Chl-*a* concentration. The y-axis is log-scaled for increased resolution. **(B)** Percentage of phaeopigments out of total sediment pigments. **(C)** cDOM absorbance coefficient at 245 nm. **(D)** cDOM E2/E3 ratio. **(E)** cDOM spectral slope from 350 to 400 nm.

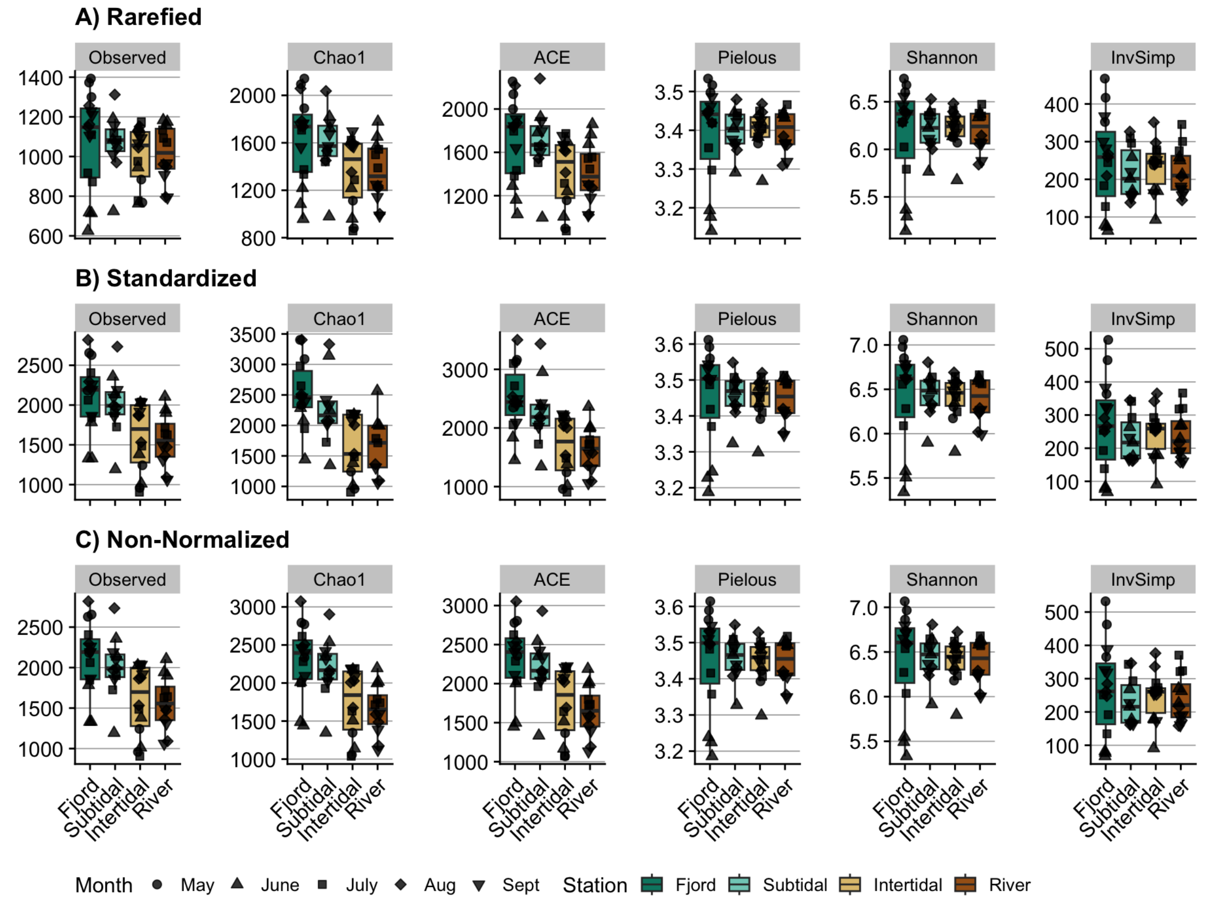

Figure S7. Boxplots displaying alpha diversity metrics for all samples from each station using **(A)** the rarefied community dataset, **(B)** the standardized community dataset, and **(C)** the non-normalized community dataset. Points represent individual samples, and their shape displays month.

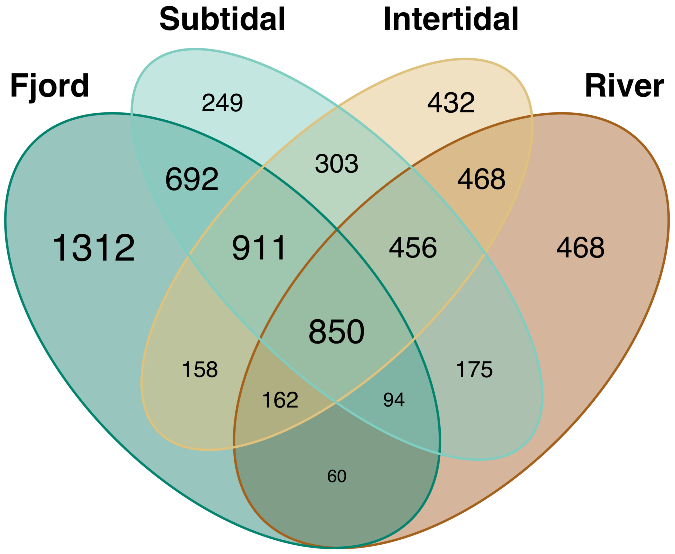

Figure S8. Venn Diagram displaying shared and unique ASVs from each station across all sampling months as evaluated on the rarefied dataset to remove artefacts of differential read counts for samples.

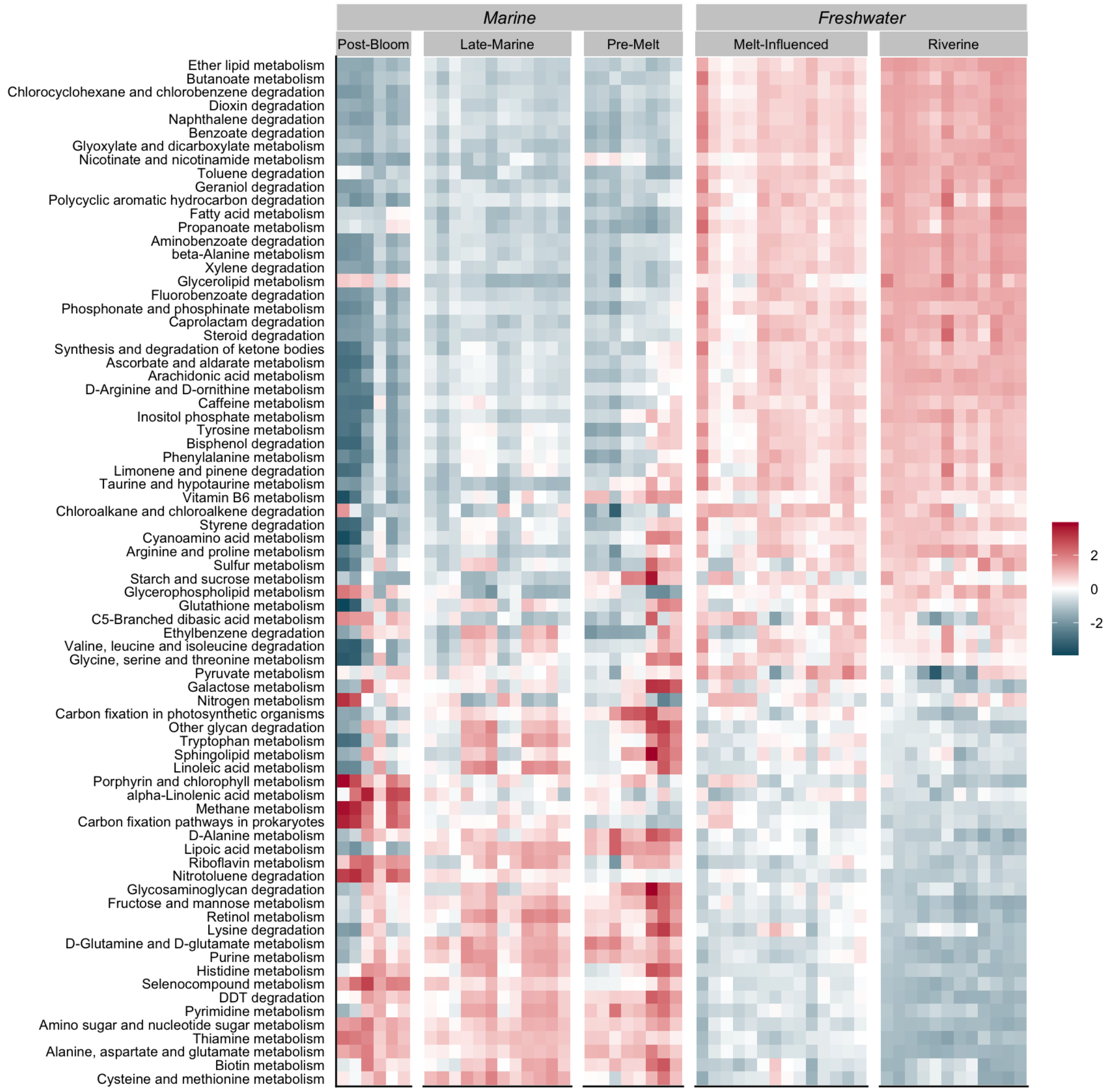

Figure S9. Heatmap displaying differential abundances of metabolic and degradation potential functional capacities predicted with Tax4Fun as based on taxonomic assignment. Potential functions were z-scaled for comparison across samples, and samples are grouped by cluster. Blue indicates a relatively low abundance while red indicates a relatively high abundance.

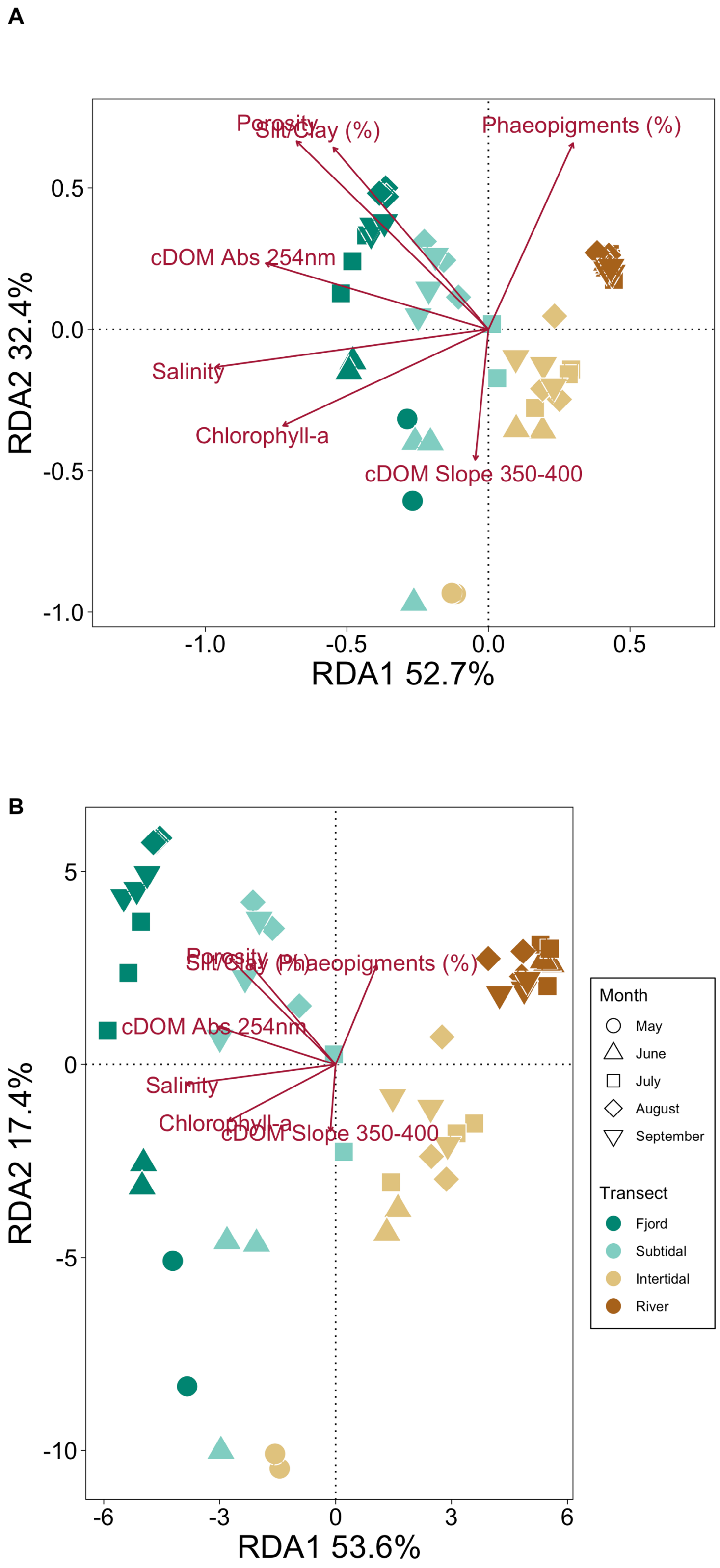

Figure S10. Redundancy analyses (RDA) on community composition and environmental variables. Community composition was **(A)** Hellinger-transformed and **(B)** centered-log-ratio (CLR) transformed prior to ordination. Environmental variables were z-scaled. The same legend applies to both figures.

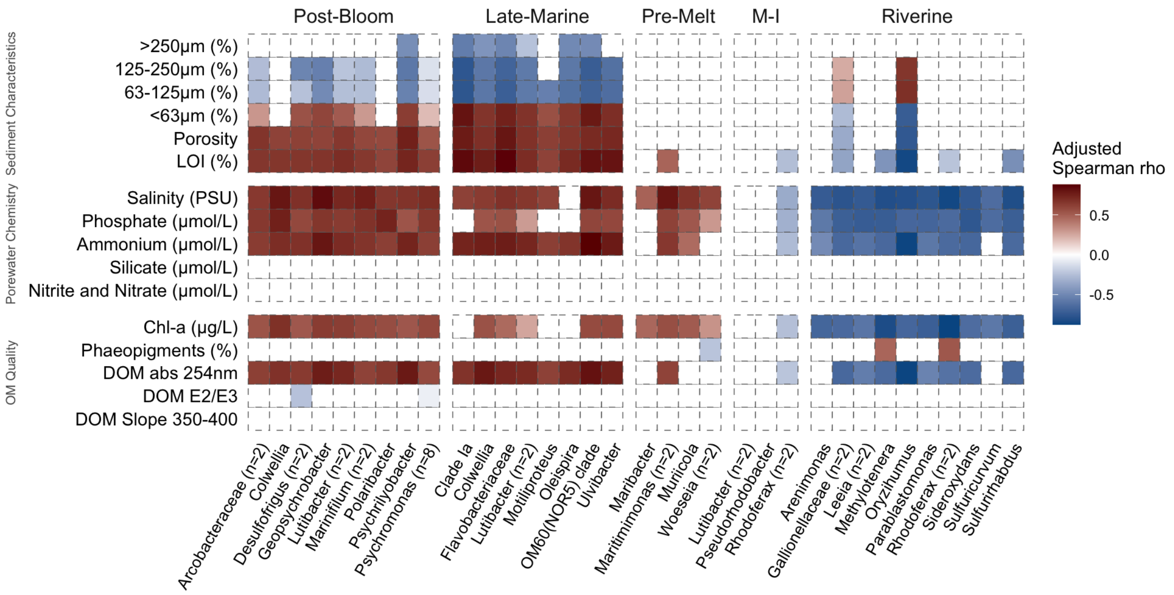

Figure S11. Spearman rank correlation of indicator taxa abundance with environmental variables, grouped by cluster (M-I = Melt-Influenced). Only correlations with an adjusted p-value (Benjamini & Hochberg, 1995) of less than 0.05 are displayed. Indicator taxa were collapsed by genus — mean spearman rank correlation is displayed is multiple ASVs were members of the same genus, indicated in the axis text. Red indicates positive correlations while blue indicates negative correlations.

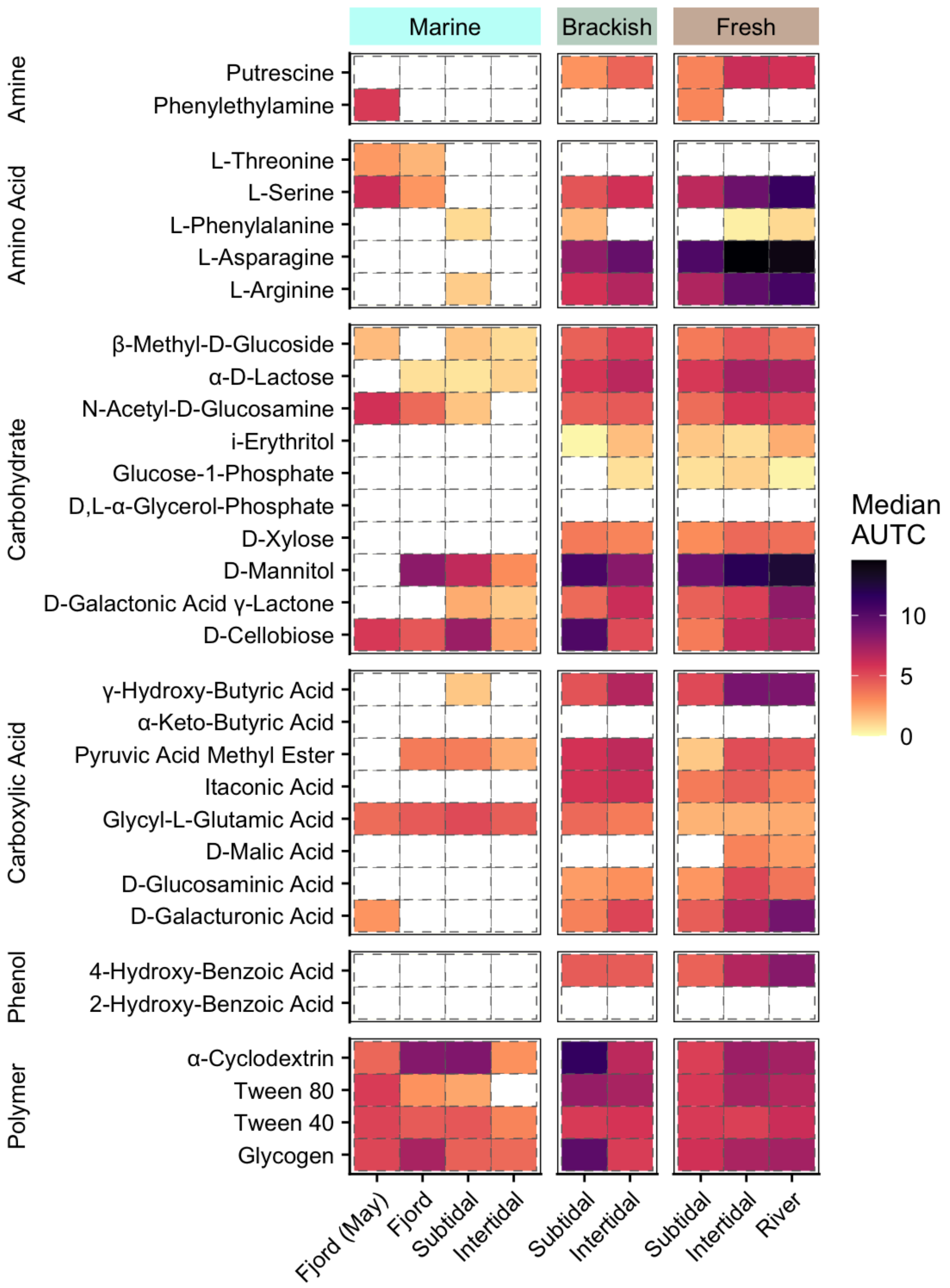

Figure S12. Heatmap displaying median area under the curve (AUTC) for each substrate on Biolog EcoPlates, with plates grouped by station and salinity of suspension water (n=4). *May* presents results from only one plate with fjord sediments suspended in deep fjord water and incubated at 4ºC for comparison. Substrates are grouped by molecular guild, according to Sala et al. (2010). Dark purple to black indicates rapid and high uptake of a substrate while light yellow indicates slower and lower uptake. Substrates that were not utilized are displayed in white.

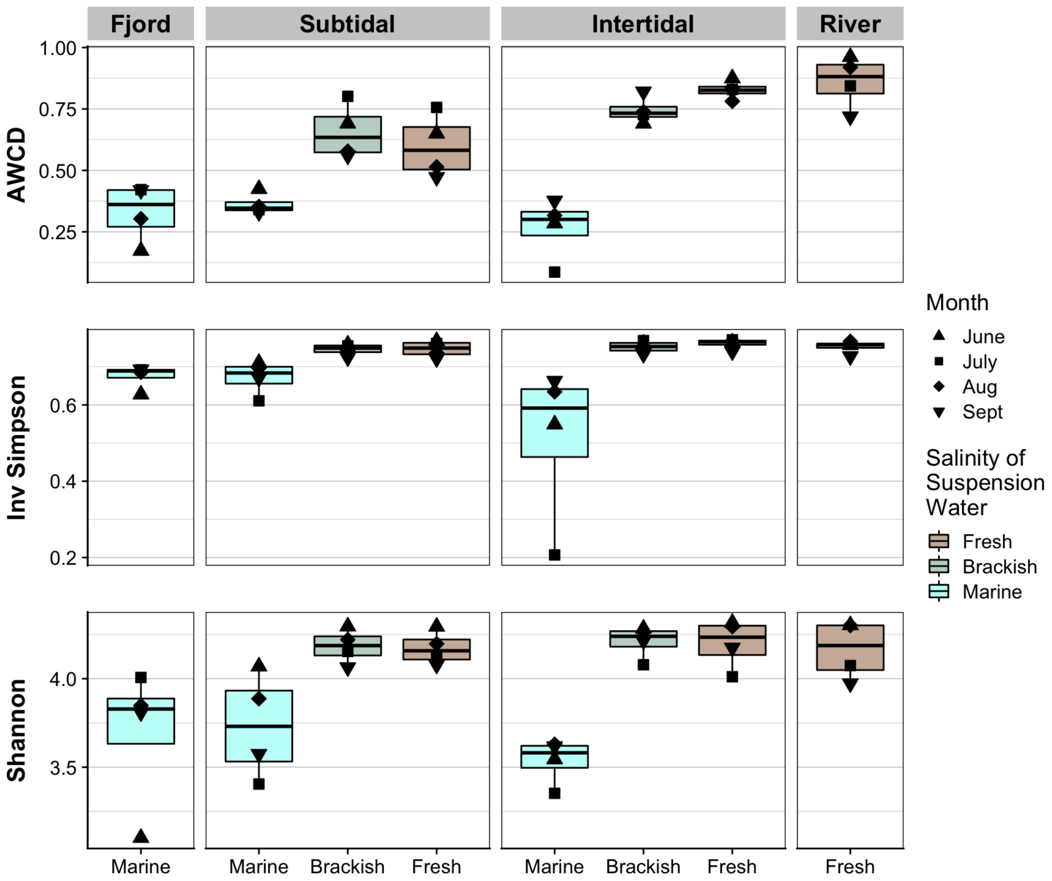

Figure S13. Boxplot showing **(A)** average well color development (AWCD), **(B)** Inverse Simpson's diversity index, and **(C)** Shannon's diversity index from Biolog EcoPlates. Plates are grouped by sediment sampling location and type of water used for sediment suspensions. Black points represent single plates, with shape depicting the sampling month.

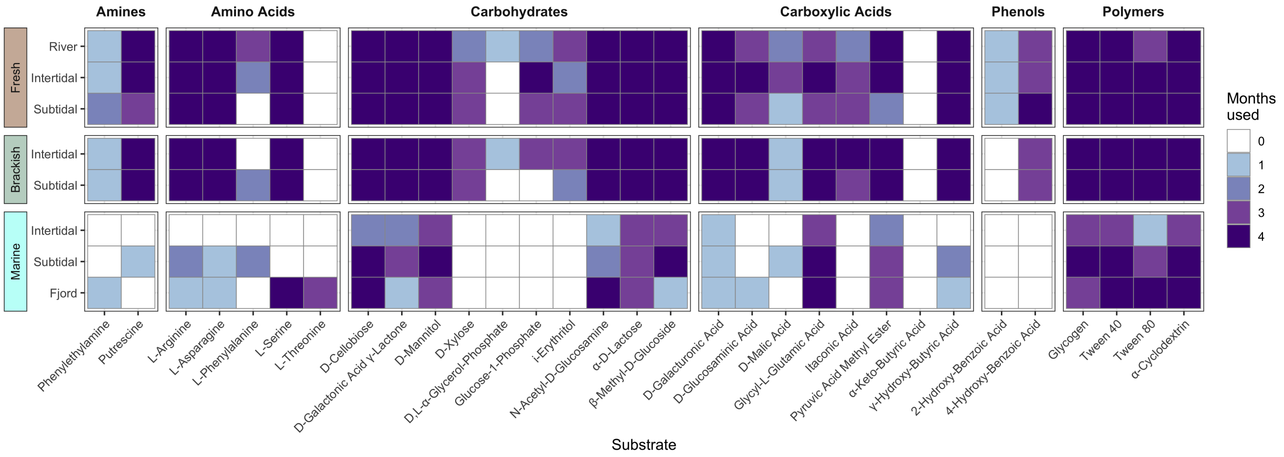

Figure S14. Patterns of substrate utilization in Biolog EcoPlates. Plates are grouped by type of water used for sediment suspensions and by sampling location. Substrates are grouped by guilds. Each cell represents the number of months a given substrate was utilized on each plate with the combinations of suspension water and sediment sampling location. E.g. phenylethylamine was utilized in one month by river sediment bacteria suspended in freshwater while putrescine was utilized all four months in the same conditions. Dark purple represents more frequent utilizations while white shows substrates that were never utilized in the conditions.

Supplemental Tables

Table S1. Sediment sampling dates, precise locations, times when sampling began, and tidal information.

Station	*Site*	*Date*	*Latitude*	*Longitude*	*Time*	*Tidal Height (cm)*	*Rising or falling?*
Intertidal	x	07/05/2021	78º13.448'N	15º41.182'E	17:28	28	Low
Intertidal	y	07/05/2021	78º13.430'N	15º41.118'E	18:20	28	Low
Intertidal	z	07/05/2021	78º13.410'N	15º40.998'E	18:35	28	Low
Fjord	x	11/05/2021	78º14.273'N	15º41.172'E	14:05	157	High
Fjord	y	11/05/2021	78º14.050'N	15º40.686'E	13:30	152	Rising
Fjord	z	12/05/2021	78º13.953'N	15º40.572'E	14:11	141	High
Intertidal	x	11/06/2021	78º13.407'N	15º42.026'E	12:30	116	Rising
Intertidal	y	11/06/2021	78º13.397'N	15º41.819'E	12:00	103	Rising
Intertidal	z	11/06/2021	78º13.419'N	15º41.678'E	11:37	94	Rising
River	x	11/06/2021	78º12.280'N	15º49.491'E	15:00	157	High
River	y	11/06/2021	78.12.269'N	15º49.448'E	14:41	157	High
River	z	11/06/2021	78º12.262'N	15º49.526'E	14:15	154	High
Fjord	x	14/06/2021	78º14.138'N	15º41.010'E	11:40	59	Rising
Fjord	y	14/06/2021	78º14.030'N	15º40.649'E	12:21	69	Rising
Fjord	z	14/06/2021	78º13.947'N	15º40.469'E	12:38	76	Rising
Subtidal	x	15/06/2021	78º13.957'N	15º41.556'E	14:05	90	Rising
Subtidal	y	15/06/2021	78º13.909'N	15º41.405'E	13:46	84	Rising
Subtidal	z	15/06/2021	78º13.854'N	15º41.243'E	13:17	77	Rising
Intertidal	x	10/07/2021	78º13.463'N	15º42.161'E	11:00	83	Rising
Intertidal	y	10/07/2021	78º13.443'N	15º42.108'E	11:22	91	Rising
Intertidal	z	10/07/2021	78º13.427'N	15º42.115'E	11:50	104	Rising
River	x	10/07/2021	78º12.287'N	15º49.452'E	14:40	150	High
River	y	10/07/2021	78º12.266'N	15º49.415'E	14:20	151	High
River	z	10/07/2021	78º12.265'N	15º49.494'E	14:01	149	High
Fjord	x	14/07/2021	78º14.188'N	15º40.896'E	14:44	112	Rising
Fjord	y	14/07/2021	78º14.072'N	15º40.630'E	15:04	122	Rising
Fjord	z	14/07/2021	78º13.957'N	15º40.564'E	15:08	125	Rising
Subtidal	x	15/07/2021	78º13.955'N	15º41.595'E	13:55	73	Rising
Subtidal	y	15/07/2021	78º13.898'N	15º41.465'E	13:35	67	Rising
Subtidal	z	15/07/2021	78º13.849'N	15º41.319'E	13:20	63	Rising
Intertidal	x	12/08/2021	78º13.410'N	15º42.042'E	13:59	100	Rising
Intertidal	y	12/08/2021	78º13.393'N	15º41.957'E	13:39	90	Rising
Intertidal	z	12/08/2021	78º13.366'N	15º41.997'E	14:30	118	Rising
River	x	12/08/2021	78º12.287'N	15º49.440'E	10:10	30	Falling
River	y	12/08/2021	78º12.274'N	15º49.467'E	10:00	33	Falling
River	z	12/08/2021	78º12.258'N	15º49.560'E	09:45	37	Falling
Fjord	x	16/08/2021	78º13.154'N	15º40.953'E	11:45	78	Falling
Fjord	y	16/08/2021	78º14.154'N	15º40.953'E	12:10	69	Falling
Fjord	z	16/08/2021	78º13.854'N	15º40.572'E	12:25	64	Falling
Subtidal	x	17/08/2021	78º13.957'N	15º41.553'E	13:55	58	Falling
Subtidal	y	17/08/2021	78º13.902'N	15º41.406'E	14:18	53	Falling
Subtidal	z	17/08/2021	78º13.855'N	15º41.240'E	14:38	52	Falling
Intertidal	x	10/09/2021	78º13.426'N	15º42.036'E	10:35	9	Rising
Intertidal	y	10/09/2021	78º13.411'N	15º42.056'E	10:15	7	Low
Intertidal	z	10/09/2021	78º13.393'N	15º41.936'E	11:00	16	Rising
River	x	10/09/2021	78º12.290'N	15º49.429'E	13:30	90	Rising
River	y	10/09/2021	78º12.269'N	15º49.444'E	13:08	78	Rising
River	z	10/09/2021	78º12.261'N	15º49.531'E	12:50	66	Rising
Fjord	x	13/09/2021	78º14.147'N	15º40.990'E	12:11	37	Low
Fjord	y	13/09/2021	78º14.044'N	15º40.692'E	12:30	37	Low
Fjord	z	13/09/2021	78º13.952'N	15º40.565'E	12:47	39	Rising
Subtidal	x	14/09/2021	78º13.954'N	15º41.527'E	13:23	45	Rising
Subtidal	y	14/09/2021	78º13.898'N	15º41.408'E	13:43	47	Rising
Subtidal	Z	14/09/2021	78º13.861'N	15º41.278'E	14:05	51	Rising

Table S2. Sediment samples used for Biolog EcoPlates. One replicate site was used for each station from June through September. In May, only fjord sediments were used and are included in this study for comparison to the melt season samples.

**Station**	**May**	**June**	**July**	**August**	**September**
River		6.River.x	7.River.y	8.River.z	9.River.z
Intertidal		6.Intertidal.z	7.Intertidal.x	8.Intertidal.y	9.Intertidal.x
Subtidal		6.Subtidal.y	7.Subtidal.y	8.Subtidal.y	9.Subtidal.y
Fjord	5.Fjord.y	6.Fjord.x	7.Fjord.y	8.Fjord.y	9.Fjord.x

Table S3. Read counts for each sample following processing steps. Bolded samples were removed from downstream analyses due to low read counts. In *Sample*, F denotes fjord, S subtidal, I intertidal, R river, and B extraction blanks.

Sample	*Input*	*Remove seq. w/ ambig. nucleo.*	*Primers clipped (remove w/ out primers)*	*Quality filter and truncate*	*Denoise Forward*	*Denoise Reverse*	*Merge*	*Remove chim.*	*Remove euk., chloro., mitochon.*	*Remove contam.*	*Remove singls.*
5-F-x	83030	66715	66274	55623	52007	53403	47059	45295	44290	44290	39429
5-F-y	67228	53873	53509	44647	41586	42688	37643	36242	35637	35637	32570
5-F-z	37625	30224	29966	24926	23339	23924	21574	20954	20086	20086	19133
5-I-x	**741**	**593**	**557**	**361**	**303**	**320**	**286**	**282**	**186**	**186**	**169**
5-I-y	26632	21362	21208	17553	16885	17081	16149	15973	13321	13321	12646
5-I-z	14527	11669	11562	9610	9124	9263	8677	8556	7743	7742	7457
6-S-x	37019	29626	29385	24711	23956	24197	23133	22866	22388	22385	21127
6-S-y	56548	45508	45188	38063	35437	36588	31371	30450	29325	29325	28373
6-S-z	59745	48117	47768	39803	37219	38173	33166	32291	31508	31508	30523
6-F-x	54020	43954	43585	35233	33767	34510	30548	28008	26861	26861	26517
6-F-y	79069	63551	63173	53251	51434	52299	47497	43401	40735	40735	40079
6-F-z	63531	50920	50593	42489	41496	42105	39541	37321	34762	34762	34299
6-R-x	71950	57727	57317	47284	44369	45179	39215	37917	37194	37194	34010
6-R-y	52779	42509	42188	34921	32644	33189	28784	27787	26922	26916	25509
6-R-z	48705	39266	38975	32429	30102	30652	26314	25405	24658	24657	23560
6-I-x	16130	12953	12838	10444	9880	9991	9169	8981	8820	8820	8476
6-I-y	22503	18149	17996	14928	13878	14166	12771	12494	12370	12370	11813
6-I-z	45397	36467	36183	30026	28158	28724	25760	25133	24824	24824	22786
7-S-x	24517	19652	19480	16306	15506	15764	14497	14205	13806	13806	13528
7-S-y	30145	24295	24079	20080	19258	19509	18173	17825	17372	17372	17059
7-S-z	**1965**	**1528**	**1478**	**1173**	**1073**	**1099**	**1005**	**988**	**972**	**971**	**925**
7-F-x	77070	62042	61583	52108	49137	50790	42753	40972	40277	40277	38715
7-F-y	89484	71972	71440	60522	57979	59418	51795	47415	44957	44957	44043
7-F-z	54998	44374	44000	36790	34899	35818	30910	29246	28389	28385	27792
7-R-x	31109	24899	24681	20324	19088	19415	17116	16549	15994	15994	15466
7-R-y	39173	31382	31153	25381	23650	24173	21078	20420	19339	19339	18392
7-R-z	40384	32494	32235	26396	25028	25272	22319	21727	21490	21490	20126
7-I-x	45355	36305	36042	29284	28224	28551	26383	25821	25007	25007	24303
7-I-y	24615	19633	19450	15831	15235	15359	14188	13948	13418	13417	13113
7-I-z	8197	6584	6508	5268	4925	5009	4492	4387	4256	4256	4130
8-S-x	51215	41017	40612	33739	32076	32783	29322	28422	28215	28214	27698
8-S-y	52303	42039	41722	34292	32699	33441	29987	29233	28818	28818	28238
8-S-z	67490	54239	53817	44864	42485	43426	39179	38207	37832	37831	36592
8-F-x	44897	36258	35934	30176	28602	29289	26106	25420	25200	25196	24619
8-F-y	75978	61215	60788	51475	49023	50019	44186	42931	42470	42470	41428
8-F-z	46483	37338	37074	31167	29832	30356	27844	27205	26941	26941	26508
8-R-x	31608	25400	25159	20804	19681	19911	17882	17411	16958	16958	16234
8-R-y	26187	21076	20853	16888	15919	16217	14340	13955	13681	13679	13257
8-R-z	18365	14865	14685	12183	11517	11677	10627	10282	9727	9727	9354
8-I-x	22185	17874	17715	14519	13830	14031	12850	12598	12180	12180	11880
8-I-y	30719	24631	24412	20317	19250	19582	17870	17378	15807	15807	15295
8-I-z	41241	33191	32844	26999	25669	26031	23892	23372	20732	20732	19404
9-S-x	43457	34838	34544	28873	27828	28256	26244	25618	25488	25488	25052
9-S-y	47028	37957	37636	31702	30417	30931	28184	27460	27311	27311	26927
9-S-z	44378	35567	35265	29565	28068	28702	25737	25167	24655	24655	24068
9-F-x	43882	35334	35063	29398	27586	28507	24094	23200	22756	22756	22311
9-F-y	30259	24293	24074	20077	18885	19465	16762	16268	16037	16037	15668
9-F-z	44694	35868	35560	29857	28265	28993	25499	24656	24361	24361	23690
9-R-x	27339	21904	21717	17914	17102	17368	15800	15388	14905	14905	14478
9-R-y	20397	16375	16212	13251	12628	12806	11673	11356	10953	10953	10650
9-R-z	33456	26837	26594	21862	21026	21203	19476	19005	18478	18478	18089
9-I-x	42417	33790	33499	27776	26829	27110	25249	24722	23350	23350	22791
9-I-y	44573	35838	35569	29699	28484	28890	26389	25748	24406	24406	23756
9-I-z	51106	40837	40482	34050	32790	33244	30817	30195	27054	27054	26079
B-1	202	145	122	34	16	7	6	6	6	NA	NA
B-2	119	93	78	16	4	3	1	1	1	NA	NA
B-3	89	62	52	17	4	3	3	3	3	NA	NA
B-4	132	107	92	36	19	20	18	18	18	NA	NA
B-5	4818	3919	3873	3157	2236	2545	1225	1225	1225	NA	NA

Table S4. Results of Dunn's post-hoc test comparing sediment characteristics between clusters. Adjusted *p*-values (Benjamini-Hochberg 1995) less than 0.05 are in bold.

Clusters		Porosity		Organic (%)		>250μm (%)		125-250μm (%)		63-125μm (%)		<63μm (%)	
		Z	p	Z	p	Z	p	Z	p	Z	p	Z	p
Late-Marine	Melt-Influenced	4.35	**0.00**	4.58	**0.00**	-2.45	**0.01**	-4.08	**0.00**	-4.65	**0.00**	4.37	**0.00**
	Post-Bloom	0.98	0.16	0.95	0.17	-2.12	**0.02**	-1.55	0.06	-2.06	**0.02**	1.69	0.05
	Pre-Melt	3.73	**0.00**	2.99	**0.00**	-3.95	**0.00**	-3.75	**0.00**	-3.12	**0.00**	3.67	**0.00**
	Riverine	4.63	**0.00**	5.00	**0.00**	-3.22	**0.00**	-3.87	**0.00**	-4.20	**0.00**	4.39	**0.00**
Melt-Influenced	Post-Bloom	-2.50	**0.01**	-2.73	**0.00**	-0.20	0.42	1.70	**0.04**	1.64	0.05	-1.78	**0.04**
	Pre-Melt	-0.02	0.49	-0.99	0.16	-1.90	**0.03**	-0.23	0.41	0.91	0.18	-0.09	0.46
	Riverine	0.46	0.32	0.60	0.27	-0.89	0.19	0.07	0.47	0.29	0.39	0.19	0.42
Post-Bloom	Pre-Melt	2.25	**0.01**	1.65	0.05	-1.37	0.08	-1.73	**0.04**	-0.73	0.23	1.54	0.06
	Riverine	2.80	**0.00**	3.13	**0.00**	-0.51	0.31	-1.61	0.05	-1.37	0.08	1.89	**0.03**
Pre-Melt	Riverine	0.42	0.34	1.48	0.07	1.07	0.14	0.29	0.39	-0.64	0.26	0.25	0.40

Table S5. Results of Dunn's post-hoc test comparing porewater chemistry between clusters. Adjusted *p*-values (Benjamini-Hochberg 1995) less than 0.05 are in bold.

Clusters		Salinity (PSU)		NH4 (μmol/L)		PO4 (μmol/L)		SiO2 (μmol/L)	
		Z	p	Z	p	Z	p	Z	p
Late-Marine	Melt-Influenced	2.78	**0.00**	4.27	**0.00**	3.31	**0.00**	2.44	**0.01**
	Post-Bloom	-1.01	0.16	0.24	0.41	-1.55	0.06	-0.37	0.35
	Pre-Melt	-1.00	0.16	1.53	0.06	-1.01	0.16	2.48	**0.01**
	Riverine	4.68	**0.00**	5.22	**0.00**	3.06	**0.00**	1.86	**0.03**
Melt-Influenced	Post-Bloom	-3.28	**0.00**	-3.01	**0.00**	-4.09	**0.00**	-2.23	**0.01**
	Pre-Melt	-3.50	**0.00**	-2.25	**0.01**	-3.97	**0.00**	0.35	0.36
	Riverine	2.08	**0.02**	1.06	0.14	-0.18	0.43	-0.54	0.30
Post-Bloom	Pre-Melt	0.09	0.46	1.00	0.16	0.64	0.26	2.34	**0.01**
	Riverine	4.83	**0.00**	3.77	**0.00**	3.90	**0.00**	1.80	**0.04**
Pre-Melt	Riverine	5.19	**0.00**	3.14	**0.00**	3.75	**0.00**	-0.82	0.21

Table S6. Results of Dunn's post-hoc test comparing indicators of organic matter between clusters. Adjusted *p*-values (Benjamini-Hochberg 1995) less than 0.05 are in bold.

Clusters		Chl-a (μg/mL)		Phaeo (%)		a254 nm		E2/E3		Slope 350-400	
		Z	p	Z	p	Z	p	Z	p	Z	p
Late-Marine	Melt-Influenced	0.64	0.26	1.64	0.05	3.95	**0.00**	1.44	0.08	0.86	0.20
	Post-Bloom	-2.82	**0.00**	2.49	**0.01**	-0.11	0.46	2.68	**0.00**	0.98	0.16
	Pre-Melt	-1.95	**0.03**	2.67	**0.00**	1.87	**0.03**	-2.29	**0.01**	-2.80	**0.00**
	Riverine	2.82	**0.00**	-0.57	0.29	5.09	**0.00**	-0.90	0.18	-0.36	0.36
Melt-Influenced	Post-Bloom	-3.40	**0.00**	1.23	0.11	-3.11	**0.00**	1.61	0.05	0.34	0.37
	Pre-Melt	-2.57	**0.01**	1.29	0.10	-1.48	0.07	-3.55	**0.00**	-3.57	**0.00**
	Riverine	2.29	**0.01**	-2.22	**0.01**	1.23	0.11	-2.35	**0.01**	-1.22	0.11
Post-Bloom	Pre-Melt	0.96	0.17	-0.05	0.48	1.62	0.05	-4.29	**0.00**	-3.16	**0.00**
	Riverine	5.11	**0.00**	-2.95	**0.00**	4.01	**0.00**	-3.37	**0.00**	-1.25	0.11
Pre-Melt	Riverine	4.47	**0.00**	-3.17	**0.00**	2.49	**0.01**	1.51	0.07	2.49	**0.01**
